# Laser-Based Fabrication of Hydrogel Scaffolds for Medicine: From Principles to Clinical Applications

**DOI:** 10.3390/gels11100811

**Published:** 2025-10-09

**Authors:** Dan Stefan Manoliu, Cristian Zagar, Irina Negut, Anita Ioana Visan

**Affiliations:** 1 Faculty of Medical Engineering, National University of Science and Technology Politehnica Bucharest, 060042 Bucharest, Romania; manoliu2003@gmail.com; 2 National Institute for Laser, Plasma and Radiation Physics (INFLPR), Atomiştilor 409, 077125 Măgurele, Romania; cristian.zagar@inflpr.ro; 3 Faculty of Physics, Research and Development Center for Materials and Electronic & Optoelectronic Devices (MDEO), University of Bucharest, Atomiştilor 405, 077125 Măgurele, Romania

**Keywords:** hydrogel scaffolds, laser-based fabrication, additive manufacturing, microfabrication, clinical applications

## Abstract

Hydrogel scaffolds have emerged as pivotal materials in regenerative medicine due to their biocompatibility, tunable mechanical properties, and ability to mimic the extracellular matrix. However, conventional fabrication techniques often lack the precision required to create complex architectures, limiting their effectiveness in tissue engineering. This review explores advanced laser-based fabrication methods, such as two-photon polymerization, laser-induced forward transfer, selective laser sintering/melting, and laser direct writing, which offer unparalleled resolution and control over scaffold geometry. These techniques enable the production of intricate 3D structures tailored to specific clinical needs, from vascular networks to patient-specific implants. We analyze the principles, advantages, and limitations of each method, highlighting their biomedical applications and the challenges of scalability, material compatibility, and cost. By bridging the gap between laboratory research and clinical implementation, laser-based technologies hold significant promise for advancing personalized medicine and tissue regeneration.

## 1. Introduction

Hydrogels are cross-linked polymers capable of absorbing large amounts of water relative to their mass, resulting in soft materials that can mimic human tissue [1]. An important property most hydrogels have is biocompatibility. Their mechanical properties and porosity are tunable by adjusting composition and crosslinking density, thus making them mimic the extracellular matrix [2]. Thanks to their mechanical properties, high water content, and favorable immune response, these materials are used more often in medical applications such as drug delivery systems, wound dressings, and three-dimensional scaffolds [3]. Their hydrated, biofriendly matrix provides support for cell and tissue integration, making them suitable for regenerative medicine.

While hydrogel scaffolds are promising, current fabrication methods like solvent casting, porogen leaching, freeze drying, electrospinning, etc., impose limitations on the achievable architecture. Conventional techniques typically yield simple or random structures caused by a lack of precise control over scaffold geometry and pore interconnectivity. This lack of precision and reproducibility may impede the formation of complex structures or patient-specific hydrogel constructs. For instance, finely creating microchannels or hierarchical porosity poses a great challenge to conventional fabrication techniques, and this in turn limits cell infiltration and nutrient transport in bulk hydrogel materials. These limitations highlight the need for novel advanced fabrication techniques capable of producing customizable hydrogel scaffolds with precise characteristics that meet modern tissue engineering requirements.

Laser-based techniques emerged as powerful tools in the fabrication process of hydrogel-based biomaterials. Advanced laser techniques offer unparalleled precision and tunability, allowing micrometer-scale resolution and precise guiding by computer control. These features aid in the creation of complex 3D architecture that was not possible with conventional techniques. Moreover, most of these techniques do not require physical contact with the synthesized material, thus leaving cells and fragile structures undisturbed. Two photon polymerization (2PP) is a type of multiphoton lithography that utilizes a tightly focused IR beam to crosslink hydrogel precursors with sub-micron resolution, yielding well-defined 3D scaffolds for tissue integration and drug delivery [2]. Likewise, laser-assisted printing methods like laser-induced forward transfer (LIFT), allow the precise deposition of hydrogel bio-ink, in a nozzle-free way, obtaining high spatial resolution and good cell viability [4]. These capabilities set advanced laser technologies apart, allowing tissue engineers to make full personalized scaffold with size scales that mimic tissue microenvironments, adapted to each patient’s needs [5].

**Figure 1:**
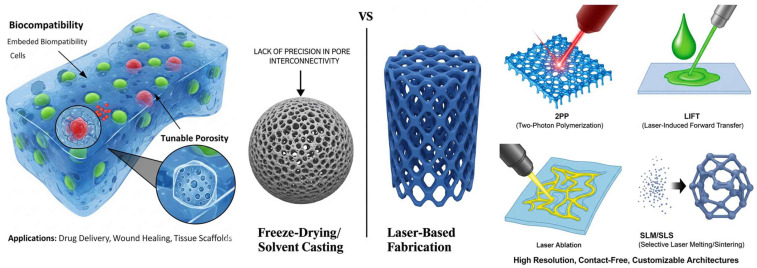
Comparison of hydrogel scaffold fabrication methods. The left panel illustrates hydrogel scaffolds created by traditional methods like freeze-drying and solvent casting, highlighting their biocompatibility, embedded cells, and tunable porosity for applications such as drug delivery, wound healing, and tissue scaffolds. A key limitation of these methods is the lack of precision in pore interconnectivity, as depicted by the solid sphere with irregular pores. The right panel contrasts these with laser-based fabrication techniques, offering high-resolution, contact-free, and customizable architectures. Specific laser-based methods shown include 2PP for creating intricate 3D structures, LIFT for precise material deposition, laser ablation for material removal, and SLM/SLS for generating complex 3D constructs.

In the following review, we present a comprehensive overview of advanced laser techniques used in the fabrication of hydrogel biomaterials, focusing on their working principles, biomedical applications, and future perspectives (Figure 1). We will analyze 2PP, also known as multiphoton lithography, which allows 3D structuring of hydrogel at micro/nano scale; laser direct writing (LDW) and laser ablation utilized for high precision modeling of hydrogels; LIFT used for laser assisted bio printing; selective laser melting/sintering (SLM/SLS), which may be used in the manufacture of solid polymers scaffolds that can be hydrated at a later stage.

Throughout this work, we will analyze these laser-based fabrication strategies, comparing the resulting resolution and biological performance, while highlighting today’s challenges and future prospects of hydrogel biomaterials obtained with these techniques.

## 2. Fundamentals of Hydrogels for Medicine

### 2.1. Hydrogel Classification

Hydrogels can be divided into three main categories, based on the nature of their constituent polymers. Table 1 provides a quick summary of the different types of hydrogels, their key characteristics, as well as their main advantages and disadvantages.

#### 2.1.1. Natural Hydrogels

Derived from biological polymers such as collagen, hyaluronic acid (HA), and alginate, natural hydrogels are highly biocompatible and biodegradable, with their degradation products being non-toxic [2,6,13]. They biochemically mimic the extracellular matrix (ECM) and often contain cell-binding sites, such as the RGD sequences in collagen, that promote cell adhesion and integration [2]. While they are generally well-accepted by the body, they can exhibit an unpredictable degradation rate and may lack intrinsic cell-adhesive ligands, which sometimes necessitates chemical modification or blending with ECM proteins [2].

#### 2.1.2. Synthetic Hydrogels

Synthetic hydrogels are synthesized from artificial polymers such as polyethylene glycol (PEG), polyvinyl alcohol (PVA), polyacrylamide (PAAm), and polylactic co-glycolic acid (PLGA), synthetic hydrogels offer enhanced control over chemical composition, mechanical properties, and durability [6,7,13,14]. Their degradation rate is more predictable and can be tuned for specific applications through modifications of polymer molecular weight, crosslinking density, or copolymer ratios [7,14]. However, they are typically bioinert and lack the cell-recognition signals present in natural polymers, leading to poor cell adhesion unless functionalized with bioactive ligands or peptides [2,14].

#### 2.1.3. Hybrid Hydrogels

Hybrid hydrogels, also referred to as semisynthetic hydrogels, combine natural and synthetic polymers to integrate the advantages of both [2,13]. Natural polymers provide biocompatibility and cell-binding sites, while synthetic polymers provide mechanical strength and structural tunability [13]. For instance, blending chitosan with polyvinyl alcohol (PVA) results in a composite hydrogel with improved mechanical performance and good biocompatibility, whereas alginate, modified with polyethylene glycol (PEG) and RGD peptides, enhances both cell adhesion and mechanical stability [2,13]. This dual functionality produces scaffolds that are simultaneously robust, biofriendly, and highly versatile for biomaterial engineering applications [13].

### 2.2. Key Properties of Resorbable Hydrogels

#### 2.2.1. Mechanisms of Degradation

Resorbable hydrogels are designed to break down in vivo into non-toxic byproducts after completing their structural or drug delivery role. Broadly speaking, there are two mechanisms through which hydrogel scaffolds degrade, namely hydrolytic degradation, characteristic to synthetic hydrogels, and enzymatic degradation, for natural polymer hydrogels [15]. Hydrolytic degradation involves water molecules diffusing through the polymer network and cleaving hydrolytically unstable bonds (e.g., ester bonds). The result is a structure which gradually degrades via hydrolysis [15,16]. This process is typically independent of biological activity and is mainly influenced by the polymer’s chemical structure and local conditions (pH and humidity) [17]. In contrast, enzymatic degradation is mediated by specific enzymes, proteases, and glycosidases, that recognize specific polymer chains [15,17]. Natural polymers, like collagen, HA, and alginate, are inherently susceptible to enzymatic breakdown. Unlike hydrolysis, which tends to be uniform, enzymatic degradation is usually site-specific [15]. In a physiological setting both mechanisms act concurrently: spontaneous hydrolysis may start the erosion of a scaffold, while the fragments or exposed sites are then cleaved by enzymes [15].

An important distinction between these two mechanisms consist in their by-products and tuneability: hydrolytic degradation rates are influenced by water uptake, crosslink density, and polymer composition, but are independent of biological variability [17]. On the other hand, the rate of enzymatic degradation is dependent on the presence and activity of site-specific enzymes, which can vary depending on the tissue or healing stages [17]. As a result, synthetic hydrogels have a more predictable and tunable time-driven degradation, whereas natural polymer hydrogels may degrade faster in enzyme-rich environments.

#### 2.2.2. Tuning to Match Tissue Regeneration

An important principle in tissue engineering scaffold design is to match the hydrogel’s degradation rate to the formation rate of new tissue [17,18]. If a hydrogel scaffold degrades too quickly, the newly developed tissue may remain without support; reversely, if it degrades too slowly, it may impede remodeling, or even generate an immune response [17]. Therefore, engineers use different strategies in order to control the degradation kinetics. The most common strategy is precise control of the polymer’s chemical composition: adding hydrolysable segments or more hydrophilic components accelerates degradation, while hydrophobic components and higher crosslink density slow degradation down through hydrolysis [17]. For example, in copolymer hydrogels like PLGA, increasing the percentage of glycolic acid makes the polymer more hydrophilic, thus giving it a higher degradation rate. Degradation time can be engineered to span a time from a few weeks to a few months simply by changing the lactide/glycolide ratio [19]. Crosslinking density and network structure also represent critical parameters for degradation control: highly crosslinked hydrogels degrade more slowly, both hydrolytically and enzymatically due to slower diffusion rates of water and enzymes into their polymer networks. At the same time, a looser network allows faster diffusion. In enzymatically degradable networks, specific cleavage sites can be added in order to tune decay rates. An example would be the use of peptide bonds in PEG hydrogels, which can be cleaved by metalloproteinases (MMPs). These types of hydrogels are stable until tissue cells release MMPs, meaning the scaffolds degrade at the same rate at which new tissue is formed [17,18]. By employing these aforementioned strategies, hydrogel scaffolds can be tailored for different tissues.

#### 2.2.3. Mechanical Properties

Mechanical resistance, as well as elasticity of a hydrogel can be finely tuned through structural design, a key parameter being the crosslinking density of its polymer matrix. Increasing it is correlated with a more rigid and mechanically resistant hydrogel [20], while a lowering it yields a softer, more elastic hydrogel [20]. By changing the crosslinker concentration, engineers were able to also finely tune its Young modulus [20]. Diversifying polymer compositions, either by blending natural with synthetic polymers, introducing crystalline phases, or forming interpenetrating/double network structures is another way to increase mechanical performance [21]. Double network hydrogels, in particular, reached compression resistance values of the order of tens of megapascals (MPa), a 50-fold increase compared to single network hydrogels. Very importantly, this brings them closer to the mechanical performance of cartilage tissue [22]. By adjusting these parameters, hydrogels can be engineered to mimic the mechanical properties of the surrounding tissues. Value ranges for the elasticity modulus in cartilage tissue is of the order of a few MPa, and hydrogels such as photo-crosslinked gelatine methacrylate can reach these values, supporting chondrocytes leading to the formation of new ECM [21]. In one study [21], an injectable double-crosslinked hydrogel was designed to modulate stiffness in situ, which, in turn, promoted the expression of cartilage-specific genes (e.g., Sox9, COL2A1) in encapsulated cells. Scaffolds used for hard tissues, like bone, require much higher rigidity. This can be achieved through the inclusion of inorganic ceramic materials, like hydroxyapatite or calcium carbonate, by mixing them in particle form with the polymer network [23]. This approach led to the rise in compression modulus, but it reduced water absorption capabilities, thus creating a trade-off between mechanical toughening and swelling capacity [23]. Nevertheless, chemically crosslinked hydrogels can be robust enough to substitute hard tissues, while softer hydrogels are more suitable for replacing and supporting soft tissue [21].

In the recent literature, it has been clearly demonstrated that laser power, scanning speed and other printing parameters can modify the microstructure of the crosslinked polymer and, implicitly, the stiffness (Young’s modulus) and other mechanical properties of the hydrogel [24]. For example, in the case of a thermoresponsive poly(N-isopropylacrylamide) (pNIPAM) hydrogel printed by two-photon polymerization (2PP), increasing the exposure dose (by increasing the laser power or reducing the scanning speed) led to an increase in the degree of crosslinking of the polymer network. This, in turn, increased the Young’s modulus of the hydrogel [24]. More specifically, a recent study showed that a micro-structured pNIPAM, printed with low laser dose had a Young’s modulus of ~150–170 kPa, while the same material printed with higher dose had a modulus of ~207–231 kPa, due to increased crosslinking [24]. Thermal expansion coefficients and other thermo-mechanical properties were also found to be sensitive to variations in laser dose [24]. Therefore, it would be worth emphasizing that adjusting the laser printing parameters (power, speed, etc.) offers an efficient method to tune the mechanical properties (such as stiffness or even thermal behavior) of the obtained hydrogel structures. This aspect is important for applications such as soft micro-robotics or 4D devices, where the material properties need to be tuned based on the desired performance [24] (Table 2).

#### 2.2.4. Porosity

The porous interconnected architecture represents a key characteristic of hydrogels, critical for tissue engineering. In the context of hydrogel-based scaffolds for tissue engineering, it is essential to distinguish between two hierarchical levels of porosity: microporosity and macroporosity. Both play critical roles in determining the biological performance and overall functionality of the scaffold. Microporosity refers to the intrinsic pore architecture within the hydrogel’s polymeric network. Microporous hydrogels allow efficient oxygen and nutrient circulation via convection and diffusion throughout the material [33]. This property is particularly important for sustaining cell viability and metabolic activity in regions distant from the scaffold surface, thereby enabling homogeneous cellular function throughout the material.

In contrast, macroporosity pertains to the larger-scale, overall pore architecture of the scaffold. High degrees of macropore interconnectivity are indispensable for efficient cell seeding, infiltration, and migration. This interconnected macro-architecture provides a continuous channel network, in which cells can colonize the entire scaffold. The structure thus allows for spatially uniform tissue formation and vascularization. A high degree of pore interconnectivity facilitates cell seeding and migration, significantly enabling cell infiltration and uniform growth of new tissue [34]. This aspect is essential because a hydrogel with isolated pores would severely hinder cell infiltration and vascularization, while a network of large interconnected pores facilitates them [21]. Hydrogels with large and interconnected pores support cell adhesion, proliferation, and new ECM production, leading to better tissue regeneration [21].

#### 2.2.5. Swelling Behavior

Hydrogels’ ability to swell is a critical property, which is closely tied to their porosity and crosslinking density. As highly hydrophilic materials, they can absorb vast amounts of water until they reach a state of equilibrium, where the internal polymer network is fully hydrated. The degree to which a hydrogel swells is known as its swelling ratio [35]. The swelling degree is inversely proportional to the crosslink density; a more weakly crosslinked network has a larger mesh size, allowing more water to be absorbed [23,35]. This property directly influences mass transport within the polymer structure and can be leveraged for controlled drug release. In drug-loaded hydrogels, water diffusion and swelling generate ways for the encapsulated drug to be released in a controlled manner [35]. Controlled release kinetics can be tuned by modifying the polymer structure and crosslinking: a faster swelling hydrogel will release the drug faster, while a slower swelling hydrogel will release its drug load in a slower, more controlled manner [35]. In general, the addition of hydrophobic components, as well as an increase in the crosslink density will slow down water diffusion and swelling, thus leading to a slower release [35].

#### 2.2.6. Biocompatibility

Biocompatibility of a hydrogel represents the capacity of the material to not generate any immune response or have any toxic effects. The material must be non-toxic and induce a weak immune response (minimal inflammation) when implanted [36]. These biocompatible hydrogels must also support cell growth, promote cell adhesion, as well as tissue integration. Natural hydrogels are inherently biocompatible because they mimic ECM and allow cells to adhere, migrate, and proliferate within them [36]. By contrast, synthetic polymer hydrogels may be bioinert and non-toxic, but lack intrinsic cell binding sites and need to be functionalized in order to avoid any inflammatory reaction [36].

Cellular interactions and bioactivity represent the way cells interact with the surface and structure of these materials. Surface chemical properties of the scaffolds can be modified to increase cell adhesion and growth factors, while drugs can be added to increase the bioactivity of the material [36]. Moreover, there are also ‘intelligent’ hydrogels which change their properties as a direct response to stimuli from biological media.

#### 2.2.7. Surface Chemistry and RGD Sequence

The presence of the RGD sequence (Arg-Gly-Asp), derived from the ECM, either on the surface or within the polymer network of a hydrogel, greatly improves cell adhesion [36]. RGD peptides bind the integrin receptors, located on the cell membrane surface, to the scaffold, thus facilitating good adhesion, and signaling pathways that stimulate cell survival and proliferation [36]. In this way, the functionalization of a synthetic hydrogel like PEG with RGD peptides changes its surface from bioinert to bioactive by promoting cell adhesion and integration in the polymer matrix [36].

Blending growth factors and drugs increases bioactivity significantly [37]. Vascular endothelial growth factor (VEGF) and other drugs are used to amplify the desired biological response and accelerate tissue healing. A hydrogel can act as a localized delivery tool for these sensitive molecules, protecting them from rapid decay and releasing them in a controlled manner. Incapsulating VEGF molecules increases their activity and favors angiogenesis [37].

Intelligent hydrogels are materials sensitive to external factors. They are engineered to modify their physical and chemical properties as a response to biological stimuli, making them suitable for on-demand drug delivery and biological sensors [36]. In particular, pH sensitive hydrogels are polymers with ionizable groups, groups which pick up electric charge, changing the amount of water inside the polymer structure. As a result, hydrogels can swell or contract depending on the environment’s pH [36]. Polyacrylic acid (PAA)-based hydrogels swell at high pH and contract at low pH, making it a suitable delivery medium for the controlled release in the gastrointestinal tract [36]. In the context of wound dressings, hydrogels can be engineered to respond to the wound’s low pH and release therapeutic chemicals.

Thermosensitive hydrogels are materials that react to variations in temperature [38]. An example of such material is a hydrogel based on poly(N-isopropylacrylamide) (PNIPAM), which has a lower critical solution temperature (LCST) of about 32 °C. Below this temperature, PNIPAM is hydrophilic and swollen, and above it the structure collapses and it becomes hydrophobic [36]. This is a valuable property that can be used to deliver cells or drugs: it can be injected as a liquid, to fill a defect and later undergo gelation in situ.

## 3. Advanced Laser Fabrication Techniques for Hydrogel Scaffolds

This section focuses on cutting-edge, laser-based technologies that have revolutionized the fabrication of hydrogel scaffolds by enabling the creation of intricate, high-resolution 3D structures with high accuracy and control unachievable by conventional methods.

Table 3 describes the principles, benefits, and limitations of the laser-based methods [1,39,40,41,42,43].

### 3.1. Two-Photon Polymerization/Multiphoton Lithography

#### 3.1.1. Working Principle

Two-photon polymerization is an additive manufacturing technique that leverages two photon absorption of near-infrared radiation (NIR). It can reach sub diffraction limit resolution within a photosensitive material [44]. Instead of a single UV photon inducing polymerization, like in classic lithography, 2PP uses a photoinitiator that almost simultaneously absorbs two NIR photons, moving from the ground state to a higher energy state than one photon would allow, and releasing free radicals that locally initiate polymerization [45]. Two-photon absorption (TPA) is a nonlinear process, so the polymerization reaction only takes place at the focal laser point within the resin, where radiation intensity reaches a certain threshold. Therefore, the volume of photopolymerization (voxel) can be contained within a very small region, at the focal point, while the radiation that crosses the bulk material has no effect in other regions [45]. This characteristic allows direct 3D writing, without the need for a mask, in any arbitrary direction inside of a photo sensible resin’s volume. Building in situ structures is thus possible at any depth, not limited to a layer by layer approach [45]. A great advantage of 2PP is that it allows voxels to have dimensions smaller than the diffraction limit. Thanks to the nonlinear intensity and polymerization threshold value, the voxel dimensions can be lower than the laser’s wavelength [45]. In practice, 2PP can have resolution of the order of a few hundred nanometers, but sub-100 nm structures have been made by using advanced techniques like stimulated depletion emission [45]. A striking example is the well-known 2PP experiment where Kawata et al. [46] built a nano bull, marking a milestone for high-resolution 2PP [45]. 2PP stands out as a micro/nano manufacturing additive technique, allowing the design of complex submicronic structures, going further than conventional methods like lithography [45,47].

From a chemical standpoint, multiphoton polymerization can be performed by cationic or radical mechanisms. In practice, for biomedical application especially, the radical chain polymerization is preferred, due to cationic initiators generating acids that may affect cells [45]. 2PP uses specialized two-photon photo initiators, type Norrish 1 and 2, chromophores with high two-photon absorption cross-sections. Once excited, type 1 initiators cleave, while type 2 initiators transfer energy to a co-initiator, both leading to the formation of free radicals [45]. These radicals target the resin’s monomer’s double bonds, leading to the formation of a 3D polymer network in a confined volume, only where the required energy threshold is reached [45].

#### 3.1.2. Materials

The photo initiator (PI) plays a key role in 2PP, since the efficiency of the process depends on their ability to absorb two photons simultaneously within the laser’s wavelength range and their ability to generate free radicals. Unlike regular PI used for lithography, a 2PP PI often has extended donor–acceptor structures to increase two-photon absorption (TPA) [45]. An example of PI is the P2Ck molecule, which has a high absorption efficiency, TPA~140 GM at 800 nm, and has been frequently used as a bio PI in recent years [45]. Although P2CK is considered biocompatible, it has been observed that it should not be used in the presence of living cells during laser exposure because it can produce toxic singlet oxygen under irradiation [45]. This is the reason why researchers are looking into more biocompatible water-soluble PI. In 2021, Zheng et al. created a PI based on carbozol, which, with the help of an ion exchange strategy, more specifically a substitution with an adequate anion, shows good water solubility and good optical properties [48]. This PI allowed the synthesis of hydrogels with minimal cell toxicity, proving the importance of water-soluble PIs [48]. In general, obtaining hydrogels through 2PP requires the PI to have high TPA efficiency, and be water soluble [48].

Regarding the prepolymers and resins normally used, 2PP evolved from rigid commercial chemicals (epoxy and acrylates) to a larger array of materials, including bioresorbable polymers and naturally derived biomaterials [45]. Initially, the first microstructures were made from standard epoxydic resins like SU8 or ORMOCOMP, proving the feasibility of 3D structuring and their limited biocompatibility [45]. Over time, researchers introduced more flexible acrylic resins (IPL 780) and synthetic hydrogels. One of the most commonly used biomaterials is polyethylene glycol diacrylate (PEGDA). It is a neutral synthetic hydrogel that is photopolymerizable and is also used for medical applications thanks to its biocompatibility [45]. Most notable, Claeyssens et al. (2009) synthesized a PCL-PEG-PCL triblock copolymer with methacryloyl end groups and demonstrated the first biodegradable 3D scaffold fabricated via two-photon polymerization [45]. Subsequently, various synthetic degradable biomaterials were successfully photopolymerized via 2PP, including star-shaped polylactides, acrylated polyurethanes, and polycarbonates, all chemically modified with methacrylate groups for photoreticulation [45]. It is worth mentioning that acrylates are more reactive in polymerization than methacrylates; however, residual, unreacted acrylate groups can be cytotoxic. For this reason, many resins intended for biomedical use prefer methacrylate compounds, which are less toxic, as evidenced in dental-grade materials [45].

**Figure 2:**
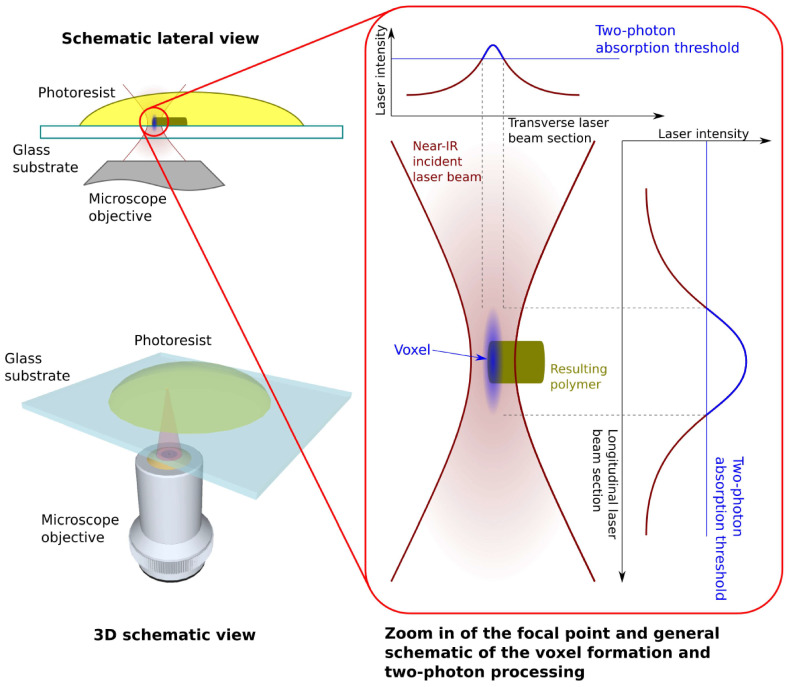
Working principle of Two Photon Polymerization, illustrating the focused laser beam, as well as the small voxel it generates, where the polymerization happens [49].

In addition to synthetic polymers, an important trend is the use of naturally derived biomaterials (collagen/gelatine, alginate, chitosan, hyaluronic acid, etc.) in 2PP, due to their favorable interaction with cells [45]. These biopolymers must, however, be functionalized with photopolymerizable groups (often methacryloyl) to make them light sensitive [45]. A successful example is gelatine methacrylate (GelMA), derived from type I collagen, containing bioactive RGD sequences. It has become a “gold standard” in the biofabrication of hydrogels. GelMA was introduced into 2PP by Ovsianikov et al. (2011), and since then, numerous studies have used GelMA to create cell-compatible, enzymatically degradable micro-scaffolds [45] (Figure 2).

#### 3.1.3. Applications

Two-photon polymerization has proven to be a versatile tool for the making of 3D micro-structures intended for biomedical applications. Due to its precision, 2PP allows the making of biomimetic architectures, closely mimicking the in vivo environment, exhibiting features down to the cellular and subcellular scale. For example, one can synthesize porous scaffolds with a precisely controlled pore size and distribution, which helps with investigating how the microstructure affects cellular behavior(adhesion, proliferation, and differentiation) [45,50]. Zang et al. (2021) demonstrated the 3D printing through the 2PP of hidrogel microscaffolds for fibroblasts, with a porosity varying between ~70–90% [50].

In the hard tissue engineering field, 2PP was used to create scaffolds that support the osteogenic differentiation of stem cells. Koroleva and colab. (2015) made 3D skeletons from a hybrid material of Zr-Si with well-defined pores (150–250 μm) using 2PP, on which they planted mesenchymal human stem cells (from bone marrow and adipose tissue) [51]. Remarkably, the cells on that scaffold showed spontaneous matrix mineralization and osteogenic markers (alkaline phosphatase (ALP), osteocalcin (OCN)) [51]. This suggests that the 3D architecture is well-controlled (for example: pores of 150 μm) and can offer mechanical and adhesion signals that are inducers of osteogenic differentiation in stem cells. Chatzinikolaidou and colab. (2015) patterned high-precision organic—anorganic scaffolds using 2PP, functionalized with BMP-2 (Bone Magnetic Protein), and have demonstrated the growth and differentiation of bone marrow stem cells on these, accelerating the formation of hard tissue [45].

#### 3.1.4. Case Studies

##### Bone Tissue Engineering: 3D Hydrogel Scaffolds That Mimic Bone Structure

In bone regeneration, a major challenge is to obtain porous scaffolds that mimic the structure of cancellous bone, providing both mechanical support and cellular diffusion inwards. Conventional porous structures often allow cell attachment only on the surface, leaving the interior unpopulated and prone to cell necrosis due to the lack of vascularization. 2PP technology has enabled the fabrication of hierarchical honeycomb microstructures with interconnected porosity and isotropy throughout the volume, designed to overcome these limitations. Păun et al. (2018) developed an innovative honeycomb scaffold using the biocompatible resin IP-L780, consisting of vertical microtubes arranged in multiple layers, with controlled free spaces between the layers [52]. By adjusting the distance between layers around 2–10 μm, volumetric migration of osteoblast cells within the 3D structure was demonstrated, which led to uniform population of the scaffold. In contrast, at distances < 2 μm or >10 μm between layers, cells could not penetrate efficiently and showed reduced matrix accumulation. Optimized scaffolds (with 2–10 μm gaps) induced strong osteogenic differentiation of cells: alkaline phosphatase (ALP) activity was ~1.5-fold higher, calcium deposits ~1.3-fold more abundant, and osteocalcin secretion ~2.3-fold higher compared to control structures [52].

##### Cartilage Tissue Engineering: 3D Scaffolds for Articular Cartilage Regeneration

Joint cartilage has an avascular structure and unique mechanical properties (high elasticity, compressive strength) that are difficult to reproduce in vitro. The goal of cartilage engineering is to develop scaffolds with appropriate porosity and stiffness, allowing for both nutrient diffusion and mechanical support for chondrocytes, in order to facilitate the regeneration of the cartilage matrix. 2PP technology has been successfully used to create microstructured scaffolds that meet these requirements. In a preclinical study by Mačiulaitis et al. (2015), researchers fabricated 3D microstructured membranes (dimensions ~2.1 × 2.1 × 0.21 mm) from a silicon–zirconium-based hybrid organic–inorganic (HOI) photopolymer, called SZ2080 [53]. These micro-scaffolds were seeded in vitro with allogeneic rabbit chondrocytes and cultured to form an adherent cell layer, after which they were implanted in vivo in rabbits, in cartilage defects, for periods of 1, 3, and 6 months. The results were promising: histological examination after explantation showed significantly improved tissue integration in the case of scaffolds with certain pore geometries and when chondrocytes were pre-cultured on the scaffold before implantation. In particular, it was observed that hexagonal pore scaffolds pre-seeded with chondrocytes favored cartilage regeneration, having comparable performance to commercially available collagen membranes (used as a reference in the repair of chondral lesions). The presence of cells at the time of implantation probably accelerated the deposition of cartilage matrix, and the hexagonal pore structure allowed for better infiltration of the newly formed tissue, compared to the grid-like geometry. In fact, the authors emphasize that the shape and size of the pores influence the way in which cartilage tissue invades and fills the scaffold: certain porous configurations led to a more organized regeneration of cartilage, mimicking the collagen structure of native cartilage. The biocompatibility of SZ2080 scaffolds was confirmed both in vitro and in vivo, with no significant adverse reactions observed; the tissue formed on the scaffolds had composition and properties similar to normal cartilage, indicating the success of the approach [53].

##### Nerve Tissue Engineering: Microstructured Scaffolds to Guide Nerve Regeneration

Regeneration of nervous tissue, especially peripheral nerves or neuronal networks in the central nervous system, requires a substrate that guides axonal growth and the formation of correct synaptic connections. The purpose of scaffolds in neural engineering is to provide physical support and topographical/chemical cues that guide the extension of neuronal processes (axons, dendrites) in an organized manner, favoring the restoration of circuits. Two photon polymerization allows for obtaining micro-structures with sub-micron features and customized geometries, ideal for interaction with neurites (neural projections). Moreover, the materials used need to be biocompatible and optically clear (transparent) to allow microscopic investigation of the formed cellular networks.

Crowe et al. developed 3D scaffolds microfabricated by 2PP with the aim of supporting the growth and neuronal alignment of neurons derived from human pluripotent stem cells (hiPSCs) [54]. In this study, two photopolymerizable biomaterial formulations were identified that demonstrated compatibility with human neural progenitor cells, allowing them to adhere, survive, and differentiate into functional neurons on the surface of the scaffolds. A major advantage of the fabricated scaffolds was high precision; the structures were created with submicron resolution and variable topographies (e.g., grooves, wires, pillars), which influenced the direction of neurite growth. It was also noted that by patterning the surface at the micron/submicron scale, neuronal processes can be guided for growth along preferential directions, forming organized 3D neural networks in vitro [54]. Furthermore, due to the optical transparency of the chosen materials and the porous structure of the scaffolds, the researchers were able to perform optical imaging (e.g., fluorescence microscopy) at individual cell level in the resulting neural network. Essentially, 2PP allowed the creation of a 3D maze for neurons, where they could be watched as they connected, while also allowing for optical interrogation (stimulation and recording) of activity at the neuronal network level, an essential aspect for the study of neurological diseases or in vitro drug testing [54].

#### 3.1.5. Advantages and Limitations

Compared to other 3D printing and microfabrication techniques, 2PP offers a number of unique advantages: extremely high resolution, true 3D volume writing capability, geometric flexibility, and compatibility with bio-applications [47]. The spatial resolution achieved by 2PP (below 100 nm, as mentioned) far exceeds conventional additive manufacturing techniques (e.g., conventional stereolithographic printing typically has a limit of ~50–100 μm). This level of precision allows the creation of microstructures with fine details at the subcellular scale, e.g., controlled roughness, channels of a few microns—which is essential for influencing cell behavior, or for making precise micro-devices. At the same time, 2PP is a contactless technology, based only on focusing a laser beam into the material. It does not require photolithographic masks, complicated subtractive development, or intermediate steps, so the design is directly transferred from the digital CAD environment to the physical structure. Consequently, the design freedom is practically unlimited: architecturally complex structures with volutes, curved surfaces, interconnected 3D networks can be manufactured, which conventional methods cannot achieve (e.g., geometries with excess undercutting, impossible to cast or engrave [47]. Another major advantage is that 2PP can use soft, biocompatible materials (hydrogels) and structure them without altering their chemical properties (the NIR laser-only interacts with functional groups at the focal point and generates radicals for ultrashort periods). Thus, living cells can be incorporated into the hydrogel (bioprinting) or can be subsequently seeded on a 2PP scaffold with fine architecture. Studies have shown that fibroblasts, stem cells, or neurons can be successfully cultured on the produced micro-scaffolds, which mimic the conditions of the natural micro-environment [50,51].

On the other hand, there are also significant limitations associated with 2PP technology, which are related to both the process and materials, as well as costs. One of the biggest disadvantages is the low production rate (throughput). Being a serial process writing voxel by voxel, line by line, 2PP is relatively slow compared to other 3D printing methods. The manufacturing time increases exponentially with the volume of the object and its complexity. For example, the fabrication of a structure of a few millimeters can take from several hours to days. Even if very high scanning speeds have been achieved (order of mm/s or even cm/s with fast galvanometers), these speeds are more valid for simple and small structures [45]. In practice, most 2PP systems write at <1 mm/s, meaning that commercial applicability to mass production is currently limited [45,47].

Another important limitation is related to cost and accessibility. 2PP equipment includes high-performance femtosecond lasers, optical systems with large apertures and very precise positioning stages (piezo nano-positioners or galvanometric scanners), which make a commercial system (e.g., nanoscribe) very expensive. The high cost of acquisition and operation (consumables, laser maintenance) has so far limited the use of 2PP more to academic and research laboratories, rather than in industry [47,55].

In conclusion, 2PP/MPL represents a technology of great finesse and versatility, capable of creating 3D microstructures impossible to achieve otherwise, with applications ranging from tissue engineering to biomedical micro-optics [47]. The key advantage is the 3D resolution and control, allowing fundamental studies on the interaction of cells with the artificial environment and the development of innovative medical devices. On the other hand, the low speed, high costs, and the limited number of photopolymerizable materials are obstacles that must be overcome to translate this technology from the laboratory to mass production or large-scale clinical applications.

A summary of the key operational parameters, material considerations, and overall capabilities of Two-Photon Polymerization is provided in Table 4.

### 3.2. Laser Induced Forward Transfer

#### 3.2.1. Working Principle

LIFT is a direct printing (writing) contactless method that uses a focalized laser pulse to eject a small quantity of material (bio ink) from a thin donor layer to a receiving substrate with very high spatial precision [63,64]. Usually, the LIFT configuration consists of a donor “ribbon”, a transparent glass substrate coated with a thin layer of bio-ink, which can be a cell-laden hydrogel, positioned above a receiving substrate. A laser pulse within the infrared spectrum is focused through the donor substrate onto the interface with the bioink layer. The laser energy is absorbed either by the bioink itself or by a thin sacrificial layer [64,65]. This local absorption generates a gas/plasma micro bubble, which rapidly expands and propels a micro droplet of ink forward, from the receiving layer to the donor layer [63,64]. Essentially, the material is transferred through a microjet formed by these droplets, that deposits precisely on the desired site. Through synchronized movement of the laser complex, models can be written point by point and through layer by layer repetition, and complex 3D aggregates can be formed [63,64].

Ideally, each laser pulse yields a single droplet with a diameter of tens of microns, allowing a micrometer scale resolution [66]. Because this technique is nozzle-free, problems regarding clogging and shear stress associated with inkjet or extrusion-based bioprinting systems are eliminated [4,63]. As such, the ink can have a higher viscosity and can contain particles or cells of any dimension without the risk of clogging a printing nozzle [63,66]. LIFT can reliably use inks with viscosity between 1 and 300 mPa·s and concentrated cell suspensions (10^8^ cells/mL), while maintaining high resolution [66]. This flexibility enabled the use of LIFT not only for live cell printing but also for the precise deposition of biomolecules and biomolecular microarrays as well as for printing microorganisms and biochemical factors with high spatial accuracy [40].

Although LIFT involves the use of a high energy laser, it is built in a way that the majority of the laser’s energy is absorbed in the support substrate (metal/polymer). More than 90% of the laser’s energy is absorbed before it reaches the cells [65]. Laser pulses have wavelengths in the infrared or visible domain, making them less damaging than UV radiation, and they are in the nanosecond region [66]. This way, cells are very briefly exposed to thermal shock and pressure, and experiments have shown that they can survive without their DNA being affected [4]. Moreover, the receiving substrate can be covered with a thin hydrogel layer that can be used as a cushion, reducing the shock when the droplet hits it. This can further increase cell viability [4]. By finely tuning parameters such as laser pulse energy, spot size, donor–acceptor gap distance, and the thickness of the bioink layer, the LIFT process can achieve stable transfer regimes, ranging from an optimal single hot mode to a high velocity or undirected multi-jet regimes [40]. In recent years, several algorithms and decision tree diagrams have been developed and published to guide the selection of laser parameters and film thickness, critical to ensuring uniform droplet formation across different hydrogel formulations [40].

**Figure 3:**
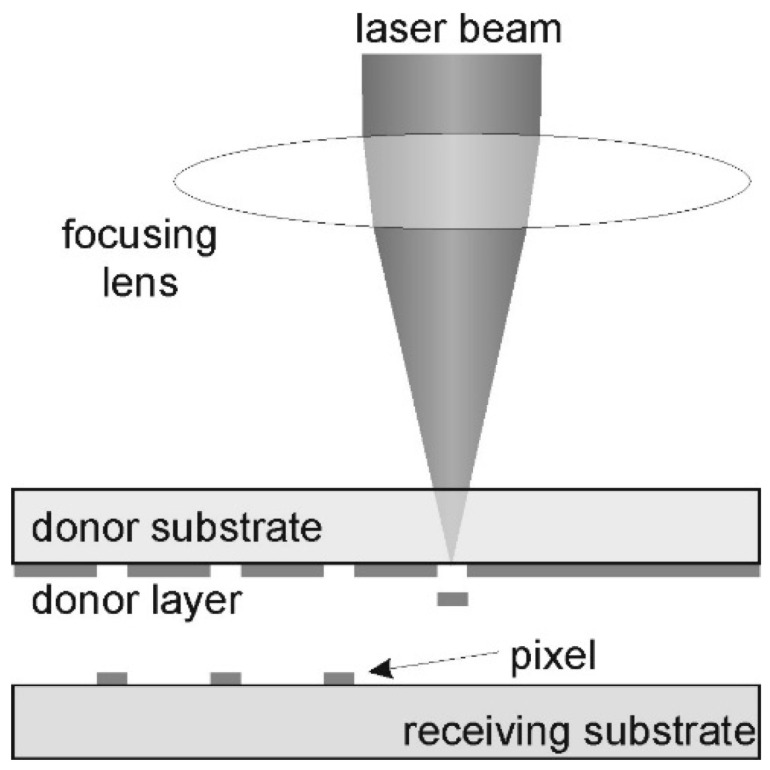
Schematic representation of the main components in a typical Laser Induced Forward Transfer system used for laser printing [63].

A great aspect of the LIFT principle is its high print speed. Each pulse forms a droplet, and the rate of droplet formation can reach several thousand pulses per second. For instance, an experimental printing speed of approximately 5000 droplets per second has been demonstrated using a 10 kHz laser combined with high-speed galvanometric mirrors [67] (Figure 3).

#### 3.2.2. Materials

A large variety of natural and synthetic hydrogels can be used as long as they can form a layer on the donor substrate. Hydrogels associated with tissue engineering, such as sodium alginates, HA, and methylcellulose, have been investigated [40].

For example, Yusupov et al., 2020, analyzed LIFT for three common hydrogels: 1% alginate, 2% HA, 1% methylcellulose, and they observed their transfer regimes from no transfer to optimal jet [40].

Type 1 collagen is another notable hydrogel material, used for creating cell suspension, successfully used for LIFT setups [64].

An example of this is the use of a collagen hydrogel matrix used to encapsulate skin cells, which are then printed using LIFT on a receiving substrate [64]. An important requirement for the hydrogel material used is a lengthy drying time. The donor layer can begin to dry after a few minutes, affecting the viscosity and jet regime. To minimize this, the donor layer should be maintained at a constant temperature and be periodically humidified. Also, hygroscopic agents like glycerin can be used; it has been observed that 10% glycerin in an alginate substrate lengthens drying time [40,68].

An important achievement of LIFT is its ability to print living cells while maintaining their viability. Typically, cultured cells are mixed into a support hydrogel at the desired density and then layered onto the donor. A remarkable aspect of LIFT is that it can work with highly concentrated cell suspensions. Successful printing has been reported at densities of 10^7^–10^8^ cells/mL, the same order of magnitude as living tissues [66]. Practically, each ejected droplet can include anywhere between a single cell and multiple aggregates, depending on the intended use [66]. Numerous cell types have been successfully printed using LIFT, demonstrating the versatility of the method. The first to show feasibility (c. 2004) had printed isolated cells one by one, such as Chinese hamster ovary (CHO) cells, without observing any impairment of morphology or survival [4]. Since then, the list has expanded substantially: human tumor cells (e.g., osteosarcoma U-2 OS), endothelial cells (bovine or human, BAEC/HUVEC), fibroblasts (mouse NIH-3T3 or human dermal) [63], neuronal cells (mouse), stem cells (adult mesenchymal, embryonic stem cells) [63], bacteria (e.g., *E. coli*) [63], algae, and yeast [63] all have been successfully transferred via LIFT [63]. Typically, imprinted mammalian cells remain viable at levels of 90–100% comparable to unimprinted controls, if the parameters are set correctly [4,40]. For example, Barron et al. reported ~95% viability immediately after printing individual cells [40], and Othon et al. (2008) showed that printed neurons did not show any function [63]. Furthermore, Las does not impose cell size restrictions: even microorganisms (bacteria, yeast) or multicellular aggregates (embryoids, spheroids) could be dispensed with without destroying them, due to the lack of shearing by the shower [63].

During and after printing, the cells remain in a moist environment (hydrogel droplet) that protects them. If proper conditions of optimal temperature are maintained, the cells remain normal and can resume proliferation in culture on the receptor substrate [63].

#### 3.2.3. Applications

LIFT is used in a series of biomedical applications that values its precision and delicacy in the placement of biological materials. Here are the present applications, with representative examples.

Micro-patterned hydrogel layers: Due to its high resolution, LIFT allows the printing of precise patterns of hydrogels on surfaces, useful for cellular-organized cultures and biosensors. An early application was the manufacturing of biomolecular microarrays, like protein spots, DNA or matrix-deposited polysaccharides on glass chips, for high-density biologic tests. Direct-wire laser technology was used to print micro-droplets (~50–100 μm) of solutions containing DNA and proteins with well-defined arrangements, introducing the concept of a contactless bio-micro-array [40].

In the context of tissue engineering, LIFT can deposit hydrogel micro-scaffolds. Catros et al., 2011 [69], used laser printing to create composite 2D and 3D hydrogel structures with bone tissue cells (osteoprogenitor cells), essentially cell “islands” in hydrogel, arranged according to the CAD design [4]. Furthermore, LIFT was combined with 2PP laser lithography in a hybrid approach: Ovsianikov et al., 2010, printed a porous polymer scaffold and then, while using LIFT, they precisely populated that scaffold with two types of cells: muscle and endothelial cells, thus gaining a vascularized in vitro tissue model [4]. This capacity to place cells or signal factors exactly on prefabricated micro-structures highlights the utility of LIFT in creating cellular micro-niches or areas of material with gradient properties. In summary, for obtaining high-resolution patterned hydrogel layers (on the order of 10–100 μm), LIFT has proven extremely efficient, allowing researchers to reproduce complex micro-patterns that guide cell behavior [67].

The bio-printing of cells for tissue growth: The most spectacular LIFT application is the tissue bio-printing, the layered construction of a tissue substitute from cells, directly in a similar configuration to native tissue. A reference example is skin bioprinting. Koch et al., 2012, performed layered arrangement of two types of skin cells (fibroblasts and keratinocytes) using LIFT to investigate the formation of dermal and epidermal layers [70]. It was found that cells imprinted in distinct layers maintained their localization and proliferation, forming a skin-like layered structure after a few days [4]. Subsequently, Michael et al., 2013 [64], managed to create a fully cellular skin substitute: they sequentially printed 20 layers of fibroblasts (in collagen), followed by 20 layers of keratinocytes on a Matriderm support [4,71]. The resulting skin graft (called “Graftskin”) was transplanted into mice, and formed stratified epithelial tissue structures after 11 days, with signs of neovascularization, demonstrating the feasibility of using LIFT in cutaneous regenerative medicine [4,71].

LIFT has also been applied in the biofabrication of blood vessels and small-scale vascular networks. Xiong et al., 2015, used LIFT to print bifurcated (Y shaped) and straight tubes from alginate hydrogel: one bioink contained plain alginate (8% *w*/*v*) for vessel walls, and the second bioink was alginate with fibroblasts (2% *w*/*v*), to introduce cells into the structures [4]. The result was the creation of micro-tubes (diameter ~hundreds of microns) populated with cells in the walls. After 24 h of in vitro maintenance, the cells in these printed vessels had a viability of over 60%, a promising result, considering the complexity of the structure [4].

However, printing very fine capillary networks remains a challenge: Koch et al., 2021, attempted to print capillaries (~tens of micrometers in diameter) via LIFT, but observed that the tubular micro-structures disintegrated within a few days due to the lack of an adequate support matrix [4].

These studies indicate that LIFT can create prototypes of small-scale vascular networks, but maintaining them stable requires either a combination with other supporting polymers or the inclusion of post-printing cross-linking processes. Even so, LIFT remains a unique tool for the directed positioning of endothelial cells, allowing research on cell–cell interactions in in vitro microvasculature models [4,63].

Another example is cardiac engineering: Gaebel et al., 2011 [72], used LIFT to create a bioprinted cardiac patch composed of two cell types: human endothelial cells (HUVEC) and human mesenchymal stem cells (hMSC). They printed these cells in an alternating pattern, on a polyurethane matrix (PEUU), essentially a cardiac patch with a defined cellular pattern, in contrast to control patches where the cells were randomly mixed [72].

After cultivation, the bioprinted patches were implanted into the infarcted hearts of rats. The results showed improved vascularization in the laser-printed patch (higher density of functional capillaries) and better functional recovery of the heart (higher ejection fraction) compared to the control group [72].

Histology indicated the integration of the imprinted human cells into the host vascular network, contributing to neovascularization. This study demonstrates the power of LIFT to organize cells into an optimized configuration (e.g., endothelial–stem pattern), which can enhance the regeneration process in this scenario, stimulating post-infarction angiogenesis. Such applications highlight the utility of LIFT in the in vitro fabrication of implantable grafts: by controlled placement of key cells (e.g., vascular cells, cardiomyocytes, pro-angiogenic factors), artificial tissues with improved functions can be obtained [72].

Multi-material and multi-cell structures: LIFT naturally lends itself to the sequential printing of multiple different materials or cell types within the same construct. Unlike single-nozzle systems, where material change is slow and prone to contamination, in LIFT it is relatively simple: multiple donor ribbons can be prepared (e.g., each loaded with a different hydrogel or cell type), which are exchanged on the platform during the process, allowing for layered deposition of distinct materials [68]. For example, in the aforementioned vessel fabrication, two different bio-inks (alginate with vs. without cells) were used in a single structure [4].

Furthermore, LIFT allows the mixing of cells with additional biomaterials, in the same structure, which expands the possibilities for applications. Keriquel et al., 2017 [73] demonstrated a concept of in situ bioprinting: they used LIFT to deposit directly into a cranial bone defect (in a mouse) a mixture of mesenchymal stem cells + collagen + nano-hydroxyapatite, layer by layer, to stimulate bone regeneration [71].

LIFT has also been used in the development of biosensors, due to its ability to deposit biological materials in a controlled manner. For example, LIFT has been demonstrated for printing DNA and protein microarrays on chips, introducing the concept of non-contact biomicroarrays [74]. Such micro-depositions have been used as sensing platforms, highlighting the feasibility of fabricating hydrogel-based biosensors by this advanced method [75]. These examples confirm the precision and delicacy of LIFT in positioning biomolecular factors, extending its applicability beyond tissue engineering and into the field of biosensors.

The prospects are significant: LIFT could be used intraoperatively to “fill” lesions with the ideal combination of cells and factors (e.g., stem cells + matrix with growth factors), directly printing a predefined design at the lesion site. In fact, LIFT has also been integrated into portable “biopen” systems, being investigated for in situ tissue repair (e.g., skin, cartilage) by layered deposition of living ink on the wound [73,76].

#### 3.2.4. Case Studies

Bone tissue engineering requires structures that mimic the layered architecture of bones and promote osteogenic differentiation of cells. The LIFT technique has proven particularly useful in the fabrication of mesenchymal stem cell-loaded hydrogel scaffolds for bone regeneration, due to its precision in arranging cells in micro-configurations that guide the formation of new bone tissue [73]. A well-known example is the study by Keriquel et al. (2017) [73], which demonstrated in vivo laser-assisted bioprinting directly in a cranial bone defect in mice, achieving accelerated bone regeneration. In this study, the authors combined a type I collagen hydrogel enriched with nano-hydroxyapatite (nHA)—to mimic mineralized bone matrix—with mesenchymal stromal cells (D1 cell line) and printed them via LIFT directly at the site of the cranial bone lesion [73].

The methodology involved a layered in situ approach. First, a thin disk of collagen-nHA hydrogel was printed at the base of the bone defect. Second, the layer of cellular bio-ink was deposited in the form of micro-droplets, according to a predefined pattern. Finally, the construct was sealed with another layer of nHA-collagen hydrogel on top. This hydrogel “sandwich” served as an osteoconductive scaffold and kept the imprinted cells confined to the bone defect throughout healing. Two different printing geometries were tested for cell distribution: a solid circular pattern (disk, diameter ~2 mm) and a hollow ring pattern (ring, diameters ~3 mm outer radius, 2.1 mm inner radius). The total number of cells deposited was the same, ~700–800 cells/mm^2^, but the spatial arrangement differed substantially between the two patterns [73].

Results: At 2 months post-printing, quantitative micro-CT analysis showed an increase in the volume of newly formed bone (BV/TV—bone volume/total volume) in defects treated with printed cells, compared to controls with hydrogel without cells; also, the disc geometry generated the highest percentage of new bone, the differences compared to the acellular scaffold being statistically significant [73].

Neural tissue engineering scaffolds: A representative study in this field is that conducted by Curley et al. (2016) [77], which demonstrated the possibility of laser bioprinting of dissociated neurons from spinal ganglia. Using the LIFT technique (also called laser direct-write in the context of that article), the researchers printed micro-droplets containing sensory neurons onto a substrate in isolated configurations, with the aim of creating spatially controlled neural nodes. The results showed that the printed neurons retained a high rate of viability after laser transfer. Moreover, the neuronal cells thus deposited exhibited extended neuronal processes—axons and dendrites—forming interconnected networks between the printed nodes. Practically, neurites grew from the printed neurons and established functional synaptic connections, demonstrating that the neural network structure can be recreated and studied in vitro by this printing method. The cells also showed normal post-printing behavior: the expected migration and proliferation were observed in the following days, similar to classical cultures, indicating that the bioprinting process did not compromise cellular dynamics. This “node engineering” experiment provides a useful model for studying neuronal interactions and has implications for the development of neural scaffolds, where populations of neurons must be strategically placed to guide the rewiring of circuits [4].

The excellent viability of imprinted cells in the neuronal context confirms observations from previous studies. As early as 2005, Hopp and colleagues reported the normal survival and proliferation of fragile cells (including neuronal lines and freshly isolated cells) after LIFT, without loss of phenotype or differentiation capacity [78].

Relevance to neural tissue engineering: The Curley case (2016) [77] demonstrates the potential for LIFT to create precisely printed, organized neural scaffolds that can facilitate axonal regeneration. The ability to place nerve cells and glial cells in precise positions means that structures such as nerve conduits can be constructed with specific gradients of cells or factors for example, sequentially printing Schwann cells in a hydrogel along the direction of the nerve, followed by printing motor neurons at the ends to promote reconnection [4].

#### 3.2.5. Advantages and Limitations

##### Advantages of LIFT

Exceptional resolution and precision: LIFT offers one of the highest printing resolutions among bioprinting techniques. Droplet size can be as low as tens of microns, or even below 10 μm in some configurations, allowing for very precise material deposition [65,66]. Patterns of detailed geometry can be printed, at the cellular, pixel level, obtaining structures impossible to achieve with extrusions (which typically have a limit of ~100 μm) [71]. For example, printing of spheroid patterns with a spatial precision of ~62 μm, smaller than the diameter of the spheroid itself, has also been reported [65]. This accuracy (order of magnitude of a cell) means that LIFT can position individual cells or small cell groups exactly where they are needed, which is essential for recreating the microstructure of native tissues.

A major advantage of laser-assisted bioprinting is that it is gentle on cells. Unlike extrusion printing, where cells undergo shear stress in a syringe, or inkjet printing, where they pass through holes and may be exposed to capillary forces, in LIFT cells “travel” freely in the expelled droplet. Numerous studies attest to viability of over 90–95% immediately upon printing [40]. Barron et al. (2005) quantified the survival of individual imprinted cells and found >95% viable, with no signs of severe stress [40]. Chang et al., 2023, note that LIFT-printed cells typically maintain 100% viability and normal growth, with no detectable functional or genotoxic alterations [4]. Even under more demanding conditions (e.g., printing large spheroids), ~80% survival was achieved without cell damage or burning [65].

Thus, LIFT is superior to many methods in this regard: it does not produce clogging, does not impose mechanical pressure on the cells, and does not require photopolymerization in the presence of cells (as in stereolithography) [4]. As a result, cell membrane integrity and phenotype are preserved. For example, it has been observed that stem cells imprinted by LIFT maintain their differentiation capacity, and neuronal or epithelial cells do not show morphological changes compared to unimprinted controls [4].

No nozzle compatibility with difficult materials: Being a non-contact, nozzle-less method, LIFT can handle inks that other printers cannot handle. Highly viscous bio-inks, polymer-rich gels, or high-density cell matrices can be printed without flow issues because there is no nozzle to manifest viscosity or particle size restrictions [63,66].

Serra and Piqué pointed out that LIFT has virtually no restrictions on particle size or viscosity, as long as the material can form a layer [63].

This aspect makes loaded inks possible: from dense cell suspensions, to hydrogels with microspheres, nano-fibers, inorganic components, which greatly expands the range of applications (e.g., printing conductive inks with metallic nanoparticles for printed electronics, or bio-inks with hard tissue components: hydroxyapatite, collagen for printed bone [63,68].

Furthermore, the absence of contact prevents contamination between materials: ribbons with different inks can be changed successively without contaminating a single extruder. Up to five different materials (using a five-ribbon carousel) have been shown to print with a single LIFT system in a single session, with rapid switching from one bioink to another [68].

Therefore, for applications requiring multimateriality (e.g., composition gradient, co-cultures of different cells, organ-on-chip structures with multiple compartments), LIFT is one of the most suitable technologies. As a computer-controlled, digital, additive process (either by moving the laser beam or the stage), LIFT has a high fidelity in reproducing the design. Print positions are programmable on a micrometer scale, and the volume of each droplet can be maintained uniform by controlling the pulse energy. High-speed film studies have shown a linear relationship between laser energy and droplet volume at a certain optimum regime, allowing precise dosing of the transferred material [40].

For example, Yusupov et al., 2020, showed that by increasing the fluence from 1 to 5 J/cm^2^, the diameter of the hydrogel droplets increases almost linearly (~100 μm at low fluences to ~300 μm at higher ones), allowing the printing resolution to be adjusted as needed [40].

Once the optimal parameters for a bioink formula are established, the process is repeatable, within tolerance. Pulse-to-pulse stability of the laser (low jitter) and maintaining a constant ink layer thickness are key factors for reproducibility [40,63].

High-throughput potential within certain limits: Although it is a “serial” technique (deposits dot by dot), LIFT can achieve impressive throughput due to the high speeds of the laser and scanner. A well-optimized LIFT system can print thousands of droplets per second [67], making it comparable to or even superior to other bioprinters in applications such as microarray fabrication or rapid cell population on a surface. Guillemot et al. mentioned LIFT among the methods with “high-throughput” potential as early as 2010, as it allows the use of lasers with frequencies of tens of kHz and fast galvanometric scanners [69].

In practice, speeds of ~10 times higher than conventional techniques have been demonstrated when printing simultaneously with an array of spots or with high-frequency pulsed lasers [79].

For example, a derived technique (HITS-Bio) has shown that ~600 spheroids can be positioned in <40 min (construct ~1 cm^3^) using similar parallel printing concepts [79].

So, at least for small-volume 2D (micro-arrangements) or 3D applications, LIFT is not a slow process. On the contrary, on a small scale it can be very efficient.

##### Limitations and Disadvantages of LIFT

Limited print volume and scalability: The main drawback of LIFT is the difficulty in rapidly fabricating large or high-volume 3D structures. Although the drop rate per second can be high, massive 3D constructs require the deposition of a huge number of drops in successive layers, which becomes time-consuming. Basically, LIFT excels at small scale (mm^3^), but it is not feasible for efficient large-scale production of bulky tissues with the current configuration [4,79].

Chang et al. explicitly state that, being limited by current printing speeds, LIFT cannot yet manufacture bioproducts on a large scale with high efficiency [4]. A similar statement is made in a 2024 study, which notes that laser bioprinting is slow and more suitable for small-sized constructs, with its use for large tissues being limited [79].

This throughput limitation means that if one wanted to print a complete organ or a large piece of tissue (on the order of cubic centimeters), LIFT would take a very long time compared to, for example, extrusion bioprinting (which can continuously deposit large volumes of hydrogel). In addition, layer-by-layer printing via discrete droplets can lead to long process times and the need to maintain sterile conditions for extended periods, which increases complexity.

Print stability and integration of printed structures: LIFT faces challenges in achieving fully functional 3D structures, especially when it comes to integrating vascularization and maintaining long-term viability at the core of the construct. For example, in the case of printed skin, it was noted that a vascular network needed to be added for the skin substitute to be viable in clinical applications [4].

Very small capillaries printed via LIFT proved unstable (dissolving in the medium in the absence of flow) [4]. Therefore, making thick, functional, and vascularized tissues by LIFT alone is difficult, most likely requiring a combination of multiple techniques (e.g., printing the outer layer by LIFT for precision, but filling the inner volume by extrusion or placing preformed vascular channels) [4].

This aspect of “thickness scaling” is an active research topic: one possibility would be sequential printing in support gel (bioprinting in a self-sustaining environment), in order to counteract droplet dispersion, or possibly the use of post-printing bioreactors to perfuse the printed structure until its own vessels develop.

Equipment complexity and optimization requirements: LIFT systems are typically more complex and expensive than conventional bioprinters. They involve pulsed lasers (often expensive), high-precision focusing optics, fine alignment of donor and acceptor substrates, and often controlled environmental chambers (to prevent drying or contamination). The cost of the equipment and expertise required to operate it can be barriers for widespread adoption outside of specialized laboratories [71].

A 2018 review notes that laser bioprinting “is an expensive process and suffers in terms of stability and scalability” compared to better established techniques [71].

Each new combination of bio-ink and cells requires calibration (optimal energy, layer thickness, distance, etc.), and there are no broadly valid standards guidelines at the moment, which means that the trial-and-error effort is significant for each new application [40].

Murphy and colleagues [80] highlighted that, compared to extrusion or inkjet, the laser method has more control parameters (energy, pulse duration, ink properties, absorber, viscosity, distance, etc.) and, therefore, optimization is more complex. At the same time, maintaining identical conditions between depositions (e.g., the exact thickness of the bioink layer) is essential, otherwise variations in droplet volume may occur.

Although LIFT can print cells at high density in a droplet, when dealing with a large 3D volume formed exclusively from micro-droplets, a diffusion problem can arise: if the droplets do not fuse completely or there is no perfusion system, the cells inside can remain isolated from nutrients. Thus, LIFT is better suited for moderately sized constructs or thin 2D/3D patterning, rather than for bulky solid entities. Nat Commun 2024 publication emphasizes that this technology is “more suitable for small-scale constructs” and that the very high cell densities required for mature tissues are difficult to achieve with laser bioprinting [79].

To achieve native cell density (hundreds of millions of cells/mL) in a printed organ, LIFT would have to print very small, very close droplets throughout the entire volume, which is difficult without compromising the viability of some cells (through possible jet overlaps or long process times in which the cells are outside the incubator).

Despite the laser pulse being largely absorbed by the donor layer, there are still some concerns regarding sub-cellular effects. An insufficiently optimized laser pulse (too short and too intense) can induce thermal stress or even DNA damage. Recent studies indicate, however, that at wavelengths around 355 nm–1064 nm and ns pulses, genotoxic stress is negligible [65,66].

In summary, LIFT brings clear benefits in bioprinting precision, versatility, and gentleness for cells, but also comes with practical challenges, such as limited throughput and scalability, higher cost, and complexity. Many research groups are trying to overcome these limitations by employing a few strategies: development of parallelized LIFT systems (with arrays of lasers or multiple spots) to increase speed, integration with other techniques (hybrid LIFT + extrusion) to combine advantages, or real-time control of printing (via high-speed cameras) to ensure the quality of each droplet [4].

As these solutions evolve, LIFT has the potential to become a standard tool in the bio-fabrication of complex tissues, especially where multiple, precisely positioned cell types and elaborate micro-architectures are required, which other techniques cannot achieve. At the moment (2025), LIFT has already established itself as a scientifically viable method, with numerous proof-of-concept demonstrations in regenerative medicine (skin [4], vessels [4], heart [4], bone [4], nerves [4]) [4].

The main challenge remaining is transfer from the lab to mass production: optimizing equipment for robustness and speed, standardizing processes, and validating the technique in practical applications. However, the research community is optimistic, as advances in automated control, biomaterials, and the integration of LIFT with multi-functional platforms could significantly expand the scope of this technology, helping manufacture personalized tissues with a level of detail and functionality unmatched by other methods [4,79].

The defining characteristics of Laser-Induced Forward Transfer, including its resolution, speed, and performance for bioprinting applications are summarized in Table 5.

### 3.3. Selective Laser Sintering/Selective Laser Melting

#### 3.3.1. Working Principle

SLS is an additive-based manufacturing technique. It uses a higher-power laser to selectively fuse the fine powder particles of the precursor material, layer by layer, following a 3D CAD model [84,85]. A thin layer of thermoplastic powder is spread on a platform, and the laser locally heats its particles above the transition temperature, fusing them without complete melting [84,86]. After selectively solidifying a layer, the platform is lowered, a new layer of powder is deposited, and the process is repeated layer by layer, until the final configuration is obtained. The entire bed of unprocessed powder acts as a support for the built layers, allowing the creation of complex structures without additional support structures [87]. The technology was originally developed at the University of Texas and is today considered one of the most established and versatile 3D printing techniques [84,88].

SLM is a similar process, also in the powder bed fusion family, but it involves the complete melting of the powder with a very high-energy laser, producing fully densified parts. SLM is mainly applied to metals that require total fusion to obtain superior mechanical properties [86].

SLM/SLS is not directly applicable for hydrogels in their typical form. These techniques rely on melting or sintering powdered materials, and hydrogels are typically water-based, soft, and easily degraded by heat, making them incompatible with the high temperatures involved in SLM/SLS.

**Figure 4:**
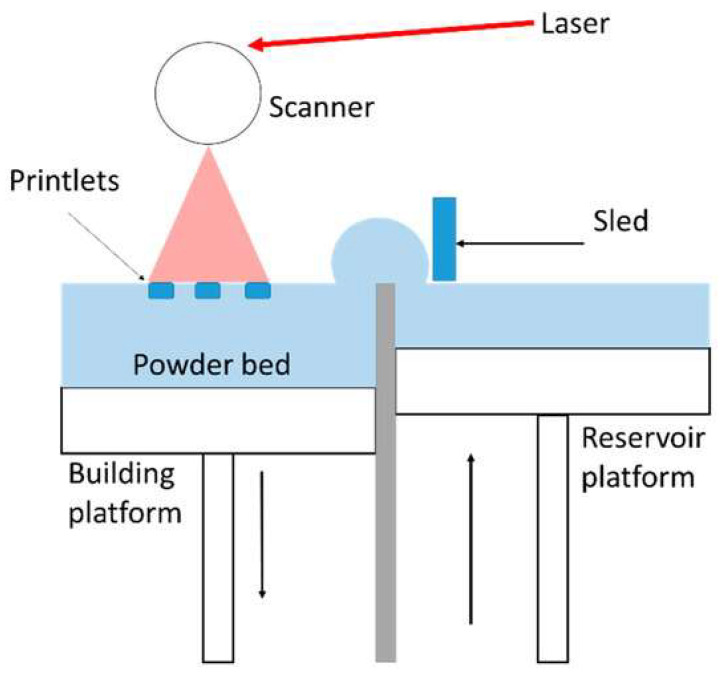
Diagram representing an SLS setup [89].

When it comes to polymers, the SLM/SLS distinction is less pronounced; thermoplastic powders are usually only synthesized to avoid degradation. However, the parameters can be adjusted to achieve almost complete fusion of the polymer wherever a higher density of the part is desired. In practice, in the field of biomedical polymers, the term SLS is used for the technique, SLM being reserved mainly for application on metal powders [86]. In principle, SLS could also be applied to polymer powders which are ultimately transformed into hydrogels. In practice, this requires special formulations, like chemical modification of the polymer to lower its melting point. A notable example is the hydrophilic polymer polyvinyl alcohol (PVA). In its pure form, it has poor thermal processability, but through molecular complexation, the hydrogen bonds are broken, lowering the melting point to ~191 °C and the crystallinity to ~28%, yielding modified PVA powder (denoted MPVA) that could be laser sintered [84] (Figure 4).

#### 3.3.2. Materials

SLS only works on materials available in dry powder form, so it is primarily limited to thermoplastic polymers, some bioceramics, and composites [84,90].

Many biocompatible polymers have been processed by SLS, for example, poly-ε-caprolactone (PCL), poly(lactic acid) (PLA and PLLA), polyamides (such as nylon-12), polyetheretherketone (PEEK), ABS, TPU [84,91].

Among these, a very popular polymer in tissue engineering is PCL, due to its low melting temperature (~60 °C), slow biodegradability (completely resorbed in 2–3 years), and high biocompatibility characteristics that make it suitable as a temporary support for bone or cartilage regeneration [86].

PCL powder has been widely used to print osteoconsolidating scaffolds; for example, Williams et al. produced porous PCL structures by SLS, whose mechanical properties (compressive modulus 10–60 MPa) are within the range of human trabecular bone [87,90].

Polylactic acid (PLA/PLGA) and its copolymers have also been experimented with, although they present challenges when it comes to sintering (narrow temperature range between sintering and degradation). Other compatible materials include polyamide-12 (PA12), widely used in industrial applications and thermoplastic elastomers (e.g., TPU) designed for flexible devices. Therefore, SLS supports a wide range of thermoplastics. In principle, any polymer that can be partially melted without decomposing can be used [91].

A valuable feature of SLS is the possibility of mixing polymer powders with solid additives (bioactive ceramics, nanoparticles) in order to obtain composite scaffolds. In hard tissue engineering, very good results have been obtained with polymer–ceramic composites. For example, PCL powders mixed with hydroxyapatite (HA) or β-tricalcium phosphate (β-TCP) print osteoconductive scaffolds, with HA mimicking the mineral phase of the bone and improving cell-support interaction [86].

Yao et al. showed that the use of a small fraction of HA (5–10% by weight) in PCL powder can increase the processability and even the resulting stiffness of the scaffold, but excessive additions (20%+) decrease the sinterability and uniformity of the component [88].

To obtain a homogeneous distribution of the ceramic in powder, special preparation methods were used, e.g., solvent evaporation in emulsion, which produced spherical PCL/HA microparticles with uniformly dispersed HA, having a good flow ability and sintering uniformly with laser [88].

Such composite powders allow the creation of scaffolds with high porosity, but also with a bioactive surface, due to the exposed ceramic.

Hydrogel precursors and special formulations:

As mentioned before, the application of SLS on hydrogel materials requires that they be transformed into thermoplastic powders.

Some examples to illustrate the concept:

By modifying PVA (to reduce hydrogen interactions and melting point), it was possible to sinter it with a laser. Li et al. used modified PVA (MPVA) mixed with 20% HA to print biocompatible scaffolds. The final pieces had a porosity of ~68% and interconnected pores, favoring osteoblast adhesion and proliferation in vitro [32].

Furthermore, an older study fabricated tetragonal scaffolds from pure PVA, with a periodic-porous structure (dimensions ~0.3–0.8 mm), by SLS. They obtained a porosity of 67.9 ± 2.7%, fully interconnected, suitable for the micro-porosity requirements of bones [31].

In vitro tests showed biocompatibility: osteoblastic cells (MG-63 line) adhere and colonize the pores of the PVA scaffold, forming intercellular bridges, which shows that sintered PVA can serve as a temporary scaffold for bone regeneration [31].

However, without modification, PVA is difficult to work with. Studies show that grinding the powder to fine granulations and carefully selecting laser power can allow sintering without chemical degradation of the polymer. Similarly, one could imagine using other polymer powders that become hydrogels through hydration or post-fabrication crosslinking. For example, polyvinylpyrrolidone (PVP) or crosslinkable polyethylene glycol could be printed as anhydrous powders and then transformed into a hydrogel. However, the available data on such experiments is very limited. Most hydrogels in bioprinting are processed by other techniques (extrusion, stereolithography) for reasons related to their chemistry (need for chemical crosslinking, presence of water, temperature sensitivity).

SLM/SLS can also fabricate entirely ceramic or metallic scaffolds, useful as rigid porous supports (e.g., metal grids for bone). Calcium phosphate powders, bioactive glass, etc., can also be sintered (often requiring a polymeric binder in the process). For example, an alveolar scaffold made of Fe_2_O_3_-doped bioglass was produced by SLS for jaw bone regeneration, the material demonstrating cytocompatibility and inducing mineralization after implantation [85].

For metals (Ti_6_Al_4_V etc.), the analogous technique is SLM, which produces fully molten structures, already used in personalized porous implants. In the context of hydrogels, these rigid materials can be considered supports onto which hydrogels can be subsequently combined (e.g., impregnation of a porous metal skeleton with a hydrogel with growth factors).

To summarize, the materials for SLS of scaffolds cover a wide spectrum of biocompatible thermoplastic polymers and their composites. To use hydrogel precursors, tuning formulation is often required, either modifying the polymer (as in the case of PVA) or adding binding agents, so that the particles can be sintered without degradation. The making of powders of appropriate size and shape is essential; usually particle sizes of the order of tens of microns, uniform distributions, and approximately spherical shapes are aimed at to ensure uniform spreading in a thin layer, as well as controlled sintering [88,90].

Strict control of these printing material characteristics is particularly important when working with hydrogel precursors, which can be more sensitive than common thermoplastics.

#### 3.3.3. Applications

The main application area of SLS in biomaterials is the production of porous 3D scaffolds, used as support for tissue regeneration. Due to high geometric freedom, SLS allows the creation of scaffolds with complex structures and controlled porosities, otherwise difficult to obtain, by conventional methods (such as foaming, lyophilization, or porous casting) [87]. For example, in hard tissue engineering, SLS has been used to produce customized trabecular structures that fit the patient’s defect, and have interconnected pores to promote vascularization and new bone growth. Williams et al. demonstrated that a PCL SLS scaffold with ~700 μm pores can support osteogenesis, providing sufficient strength for small bone defects [87].

For larger defects, PCL scaffolds prepared by SLS were reinforced with bioactive ceramic (HA), enhancing osseointegration. Wiria et al. (2008) fabricated PCL/HA scaffolds, showing that HA does not compromise the SLS process, while the resulting pieces support cell adhesion and mineralization [92]. Chen et al. (2017) [93] created gradient scaffolds (osteogenic and chondrogenic zones) through SLS, with a hierarchical structure inspired by osteocartilage, which, when implanted in rabbits, repaired osteochondral lesions [90].

Overall, both PLLA and PCL fabricated via SLS have demonstrated feasibility for various tissue engineering applications, providing customized porous structures according to the requirements of the target tissue [87].

A disadvantage of SLS is that the process itself cannot directly print cells (due to the high temperatures involved). However, the resulting porous scaffolds lend themselves very well to subsequent cell seeding or impregnation with bioactive hydrogels. A common approach is a post-fabrication combination. A rigid, porous scaffold is printed by SLS (e.g., from PCL, PLA or composite), then the pores are flooded or lined with a hydrogel containing cells, growth factors, or other biomolecules. Chen et al. [93] did this for cartilage; they seeded chondrocytes in a collagen gel, then loaded it inside the SLS scaffold made of PCL, obtaining a hybrid construct. Their studies show increased chondrocyte proliferation and deposition of cartilaginous matrix (collagen II, proteoglycans) inside the printed scaffold [87].

Similarly, the injection of chitosan hydrogels into PCL SLS scaffolds for bone applications has been reported, giving them a more hydrophilic surface and promoting cell colonization [90].

Hydrogel coatings can also be applied. For example, coating the surface of SLS scaffolds with a thin layer of hydrogel, including adhesion peptides (RGD) or ECM proteins (collagen, gelatin) is possible. A study showed that coating PCL scaffolds with a composite hydrogel of HA and gelatine significantly increased bioactivity; mesenchymal stem cells produced more GAG and were better differentiated chondrogenically on these hydrophilic supports, as compared to uncoated scaffolds [90].

Another interesting variation is the inclusion of biomolecules (proteins, growth factors, drugs) in the powder material used for SLS, in order to obtain a controlled release device. Directly, the high temperatures in SLS would inactivate most delicate biomolecules, but possible solutions, such as their microencapsulation, have been proposed: Duan and Wang (2015) fabricated polymeric microspheres that encapsulated osteoinductive proteins, then used these microspheres as powder for SLS, to build the scaffold [87].

Although the encapsulation efficiency was ~24%, the study demonstrated the feasibility of integrating growth factors into the printed material, partially protecting them from heat and allowing their gradual release in vivo [87].

Another approach was surface selective sintering: Bai et al. mixed PLA powder with carbon black nanoparticles. By irradiating with infrared laser, only the carbon absorbed the energy, melting only the surface of the neighboring PLA particles, superficially fusing them [90]. Thus, the interior of the PLA particles (which could contain a drug or a thermosensitive protein) remains relatively cool, surviving the process, while the particles bond together at the periphery. This technique opens up the possibility of making pharmaceutical tablets or drug-delivery supports directly by SLS, their high porosity facilitating rapid dissolution or controlled release of active substances [89].

Moreover, in the pharmaceutical field, it has been highlighted that the key advantage of SLS over FDM or stereolithography is precisely the obtaining of internally porous shapes without additional effort, which allows the formulation of oral doses with ultra-fast disintegration [89].

In conclusion, SLS applications, in combination with hydrogels, are mainly performed sequentially. Initially, the structural scaffold is printed by SLS, from a compatible material (PCL, PLA, composites). Then the hydrogel component (which may contain cells, biological factors) is added by impregnation, incorporation, or coating.

#### 3.3.4. Case Studies

A good example of PCL + collagen scaffolds made by SLS for cartilage tissue is the porous polymeric scaffold impregnated with cellular hydrogel—it is provided by the research of Chen et al. [93] (Chang Gung Univ., Taiwan). They fabricated poly-ε-caprolactone (PCL) scaffolds with controlled architecture by SLS (e.g., fiber deposition patterns at 0°/90° or 0°/45°/90°/135°, generating pores with different shapes and distributions) [94]. The resulting scaffolds had interconnected porosity, specifically designed to support joint cartilage regeneration. To enhance interaction with cartilage cells (chondrocytes), the team loaded these PCL scaffolds with a type I collagen hydrogel populated with autologous chondrocytes, thus obtaining a PCL/Collagen hybrid construct [94].

The results showed significant benefits of the presence of collagen hydrogel in the scaffold. Cell culture on these hybrid constructs revealed better chondrocyte proliferation when compared to PCL scaffolds without hydrogel, as well as an increased production of specific cartilage matrix. After 3 and 4 weeks in vitro, collagen, respectively, gel-loaded scaffolds showed significantly higher synthesis of glycosaminoglycans (GAGs) and type II collagen (a marker of hyaline cartilage), compared to bare PCL scaffolds [95]. The hydrogel batch had a total collagen content ~3 times higher than the batch without hydrogel, indicating a stimulation of the chondrogenic phenotype of the cells due to the hydrated and protein-rich microenvironment provided by the gel [95]. Scanning electron microscopy and confocal microscopy observations confirmed a more uniform distribution of cells and matrix inside the PCL/collagen scaffolds, compared to cells cultured on PCL only surfaces [94]. Furthermore, biomechanical tests (dynamic mechanical analysis) showed that incorporating the hydrogel enhances the construct’s ability to absorb shock waves, thus approaching the viscoelastic behavior of natural cartilage [94].

#### 3.3.5. Advantages and Limitations

SLS can produce porous 3D structures with very complex architectures, tailored precisely to one’s needs (e.g., gradient porosity, internal channels, customized anatomical shapes), something which is difficult, or even impossible, to achieve using conventional manufacturing methods [87]. Unlike FDM or SLA, SLS does not require dedicated support structures, as the uncured powder supports the top layers during deposition, so internal cavities and maze-like networks can be printed without any problem [87]. SLS parts automatically come out porous, with interconnected pores, due to the incomplete fusion of the powder: there are always spaces left between the sintered particles, which gives the structure low compactness and, therefore, the necessary porosity for biomedical applications [85]. The degree of porosity is directly influenced by the manufacturing process. For instance, using lower laser energy or higher scanning speeds will only partially sinter the material, creating a more porous structure, with a greater number of open pores. In contrast, using higher-energy parameters will cause the material to become denser, which can reduce porosity by closing pores) [89]. The possibility to modulate internal porosity at will is a huge advantage. In tissue engineering, one can adjust the porosity to obtain the optimal tradeoff between space left for cell invasion and mechanical strength of the scaffold [89]. As a concrete example, SLS scaffolds with ~40% porosity were manufactured, which proved ideal for uniform seeding of stem cells and bone formation in vivo, compared to variants with 20% and 80% porosity [86]. The ability to create porous structures without a secondary process is a key advantage of SLS over other 3D printing technologies. While FDM lays down solid filaments (requiring deliberate design for any voids) and SLA yields dense parts that must later be perforated or treated with porogens, SLS directly produces spongy, interconnected pore networks. This unique capability, which allows for pores ranging from tens to hundreds of microns, makes SLS an ideal method for creating scaffolds for tissue engineering [87].

Many materials can be used in SLS without the need for the polymerization of additives or catalysts. In photopolymerization (SLA), chemical photoinitiators are required, often toxic or with residual cytotoxic potential in the part. SLS, on the other hand, consolidates the powder physically, through heat, so it does not require the presence of chemical bonding agents, a clear biomedical advantage. Sintered parts contain only the base material, without an initiator or solvent residues [90]. For example, a PCL scaffold produced by SLS will contain only PCL (possibly with minimal traces of thermal oxidation), whereas a resin-printed SLA scaffold involves monomer polymerized in situ using an initiator, traces of which may remain in the material and affect cell viability. SLS avoids this problem and is thus considered a method without additional chemical reagents, friendly for biological applications [90].

Polymer parts made by SLS typically have microscale roughness surfaces, due to partially melted powder particles that form granular relief. This microscale roughness is advantageous for cell adhesion and proliferation: cells attach more easily and anchor more firmly to rough surfaces than to completely smooth ones [90]. Studies on SLS scaffolds made of PCL have shown better osteoblast adhesion, as compared to smooth cast surfaces made of the same polymer, precisely due to the rough topography that mimics the asperities of the natural ECM [90].

The main disadvantage of SLS for bioactive applications is that the process operates at high temperatures (typically 100–200+ °C for polymers, and much higher for metals). This means that living cells or sensitive growth factors cannot be printed directly; any biological component introduced into the powder would be locally exposed to temperatures that would destroy cells or degrade proteins [84,90].

While SLS is a promising technique, it is not universally compatible with all materials, particularly those used for hydrogels. The process demands fine powders that possess excellent flow properties and thermal stability. For polymers that form hydrogels, meeting these requirements can be difficult, as the typical methods for creating fine powders often introduce complications.

The most common approach, milling, produces particles with irregular shapes and a broad size distribution. These features can cause the powder to clump together (agglomerate) and settle unevenly, leading to defects in the final product. The ideal way forward is to use spherical powders, which are typically created through more complex processes like atomization or controlled precipitation. However, these methods are often expensive and may involve toxic solvents, making them impractical for some applications. To overcome the poor flow characteristics of irregularly shaped powders, additives such as fumed silica (a “flow agent”) are sometimes used. These agents help ensure the powder settles into a thin, uniform layer, which is essential for the success of the SLS process [90]. Compared to stereolithography or electrospinning, SLS does not excel at micro-detailing below ~100 μm. The minimum feature size is limited by the laser spot diameter (typically 200–500 μm for CO_2_ lasers) and the powder particle size (around 50–100 μm). As a result, very sharp edges, corners, and details below ~0.5 mm are smeared, or cannot be reproduced faithfully [85].

In conclusion, SLS offers remarkable advantages for the fabrication of porous scaffolds, such as complex architecture, intrinsic porosity and interconnectivity, compatibility with numerous biocompatible polymers and composites, all without the need for toxic chemicals. Due to these attributes, SLS has been called “the most popular laser rapid prototyping technology in biomedical engineering”.

The technical specifications and defining advantages and limitations of Selective Laser Sintering are summarized in Table 6.

### 3.4. Laser Direct Writing

#### 3.4.1. Working Principle

LDW refers to the use of a focused laser beam to locally modify a hydrogel, either by adding material (via photopolymerization) or, more commonly, removing material (ablation), to create desired microstructures [99]. In the case of laser ablation (subtractive method), laser energy breaks chemical bonds in the polymer network of the hydrogel and vaporizes or removes the selected volume, allowing for precise cutting, surface carving, or the creation of microchannels in preformed hydrogels [99]. The process can be performed on thin films of hydrogel, or in the bulk of a hydrogel, as long as the material is sufficiently transparent to the laser wavelength to allow it to focus inside [101]. By controlled displacement of the beam (or the sample) according to a predefined pattern, it is possible to generate microstructures with micron-level resolutions [99,101]. For example, focusing high-power pulsed lasers into a transparent hydrogel enabled punctate photoablation with sub-cellular dimensions, creating 3D-directed microchannels [101]. In comparison, additive methods (photopolymerization with a laser in a liquid precursor medium) can produce complex geometries but are limited to photosensitive hydrogels; in contrast, laser ablation works directly on already crosslinked hydrogels, offering versatility in processing [99]. The fundamental mechanism of laser–hydrogel interaction involves the absorption of photons by the material, followed by local heating, bond destruction, and material expulsion (by vaporization or bubble formation) into the interaction zone [93]. To obtain well-defined micro-structures, the laser parameters (wavelength, pulse duration, pulse energy, scan speed) must be optimized according to the hydrogel properties [93]. Typically, short-pulse lasers (pico- or femtosecond) are preferred, because they deliver highly concentrated energy in an extremely short amount of time, minimizing heat diffusion and out-of-focus thermal damage [102]. Thus, ultra-short pulse laser ablation can produce fine cavities and channels, with well-defined edges, even in soft materials, all with minimal damage to the surrounding structure [102,103]. An illustrative example is UV laser photoetching of the surface of a gelatin hydrogel: by photosensitizing the gelatin with riboflavin-5′-phosphate, a non-toxic photosensitizer, the researchers ablated a pattern of microstriations (grooves and pillars) 10–30 μm wide, without compromising the mechanical properties of the hydrogel [102].

**Figure 5:**
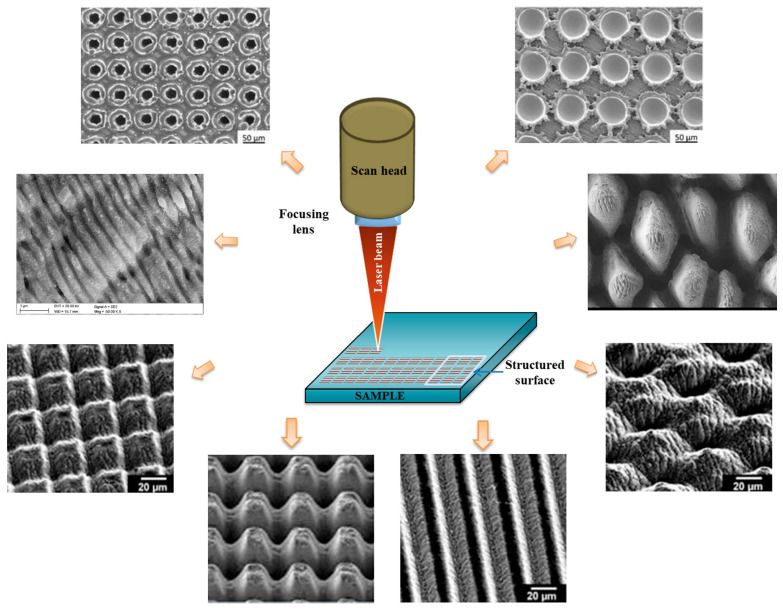
Example of laser-induced surface structuring, accompanied by SEM micrographs of the resulting features [104].

To summarize, the principle of LDW/laser ablation consists of the direct, mask-free writing of microstructures by selectively “burning” the hydrogel in the areas targeted by the beam, thus obtaining personalized patterns at the micrometric scale (Figure 5).

#### 3.4.2. Materials

A wide variety of hydrogels can be microengineered by laser ablation, from natural to synthetic hydrogels. Natural, protein-based hydrogels (e.g., gelatine, collagen), were successfully laser patterned.

In the aforementioned example, gelatine hydrogels were photoetched using a UV laser (355 nm), after impregnation with riboflavin, thus demonstrating reliable formation of microgrooves without burning or forming bubbles, provided gelatine is properly photosensitized [105].

Synthetic hydrogels, like those made from PEG, were also used: Sarig-Nadir et al. used pulsed lasers to trace microchannels in PEG fibrinogen hydrogels, obtaining guidance structures for nerve cells, directly within the hydrogel volume [101].

Additionally, acrylic hydrogels (e.g., PAM) are compatible. In combination with absorbing additives (e.g., nanoparticles), they can be efficiently ablated with femtosecond lasers [106].

Conductive, composite hydrogels represent another category: Park et al. incorporated graphene oxide into polyacrylamide (GO/PAAm) to create a conductive hydrogel, then used femtosecond laser ablation to shrink linear micropatterns on the surface [106].

Patterns (lines spaced 20–80 μm apart) were clearly formed on the GO/PAAm hydrogel, demonstrating that hydrogels containing nanomaterials can also be laser processed with high resolution [106].

Similarly, conductive polymer hydrogels (e.g., PEDOT:PSS hydrogel) were cut with laser, obtaining flexible micro-electrodes and achieving a resolution of ~6 μm for the channels, in a hydrogel encapsulated in PDMS [99].

Photosensitive hydrogels (e.g., methacrylate precursors—GelMA, PEGDA) can be directly photopolymerized by laser (additive techniques), but for subtractive LDW, it is often necessary for the hydrogel to have absorption at the laser wavelength. In the cases of laser-transparent materials, dyes or photosensitizers can also be added: for example, the aforementioned riboflavin acts as a UV photo-acceptor, allowing uniform ablation of gelatine at 355 nm, without carbonization [105].

Laser ablation on rigid hydrogels (such as polycaprolactone, hydroxyethyl methacrylate—PHEMA contact lenses) has been demonstrated: a continuous CO_2_ laser was able to etch microchannels into hydrogel lenses, albeit with some limitations, while subsequent use of femtosecond lasers on lenses produced much more precise channels (~80 μm widths) and better defined contours [103].

Therefore, any cross-linked hydrogel, whether natural, synthetic or hybrid, can, in principle, be laser-patterned, provided the right conditions are chosen. Studies show that femtosecond lasers can efficiently process a wide range of hydrogels, compensating for the limitations of traditional methods, and allowing the introduction of ECM-like micro or nano-structures into these soft materials [107].

#### 3.4.3. Applications

LDW/laser ablation offers the possibility of creating functional microstructures in hydrogels, useful in biomedicine and tissue engineering. One of the most important applications is the fabrication of microfluidic channels in hydrogels for Lab-on-a-Chip devices, or implanted sensors. For example, using a CO_2_ laser and, later, a femtosecond laser, researchers have fabricated microfluidic contact lenses with channel networks etched directly into the lens’s hydrogel material, allowing tears to be directed to certain areas, to detect ocular biomarkers [103].

A study reports the successful UV laser etching of amicrofluidic channel, fully integrated into a soft contact lens (commercial PHEMA hydrogel), including an inlet, a reaction microchamber (with fluorescent detection probe), and a reservoir, the latter used to monitor the concentration of ascorbic acid in tears, a potential indicator of ocular inflammation [103].

A second major category of applications is the micropatterning of hydrogel surfaces for cell guidance and tissue engineering. Microgrooves, ridges, or pillars ablated on the surface of a hydrogel can influence the orientation and behavior of cultured cells. For example, topographic guides for neurons have been created by laser photoablation in 3D fibrinogen–PEG-based hydrogels. Channels, a few microns in diameter, drawn in the hydrogel volume, directed the extension of axons from dorsal root ganglia, demonstrating the potential of these structures in nerve regeneration [101].

Similarly, skeletal muscle cell alignment was improved by laser microstriations on a conductive composite hydrogel; a pattern of parallel lines (20–80 μm spacing) was patterned with femtosecond lasers, on a polyacrylamide hydrogel with graphene oxide, which led to superior alignment and maturation of C2C12 myoblasts, relative to unpatterned substrates [106].

This micropatterned hydrogel also allowed for efficient electrical stimulation of cells (due to its conductivity), highlighting applications in the development of active scaffolds for muscle engineering [106].

Laser ablation is also used to define internal geometries of scaffolds, designed to mimic vascular networks [102]. By 3D scanning a laser in volume, networks of interconnected microchannels can be produced in hydrogels, serving as a template for vasculogenesis, or perfusion in 3D cultures. A recent study demonstrated that a femtosecond laser, applied sequentially to a pre-dried hydrogel (for temporary stiffening), can etch micro-grooves on the surface, while simultaneously modifying the adjacent volume (via lower laser doses), thus creating a “multilayer informational” hydrogel. Its structural channels, optical indicators and dynamic properties are used for encoding and displaying multiple bits of information in a single material [102].

In the field of bioelectronics, laser ablation of hydrogels allows the integration of soft components into devices. For example, microfabricated electrodes have been patterned onto conductive hydrogels to create implantable neural interfaces. Won et al. (2022) [108] fabricated electrodes from PEDOT:PSS hydrogel. Finely laser-cut, and then encapsulated in elastomer for stimulation and monitoring of the sciatic nerve, it achieved a soft, conformal contact, which caused minimal damage to the nerve tissue when compared to rigid metallic electrodes [99].

#### 3.4.4. Case Studies

##### Case Study 1: Neural Tissue Engineering—Photoablated Microchannels to Guide Nerve Regeneration

Regeneration of severely damaged peripheral nerves remains a challenge, despite the use of nerve conduits. In practice, autologous nerve grafting is still the gold standard, as current artificial conduits do not achieve the same efficacy in restoring function [101].

Research indicates that the success of nerve regeneration depends on guiding axons in the correct direction and creating a microenvironment that supports the migration of glial cells and neurons along the injury pathway [101].

To introduce topographic guidance cues inside a 3D hydrogel (mimicking the extracellular matrix), Sarig-Nadir et al. (2009) developed a focused laser photoablation method to create microchannels in biosynthetic hydrogels, demonstrating control of axonal growth direction in three dimensions [101].

In this study, the authors used a hydrogel composed of PEGylated fibrinogen (a bioactive semisynthetic matrix) seeded with dorsal root ganglion explants (an ex vivo model containing sensory neurons and glial cells) [101].

By applying focused laser pulses (nanoseconds or femtoseconds) inside the transparent hydrogel, they induced local photoisolation of the solid volume of the gel, creating cylindrical channels with a diameter of the order of microns [101].

These microablations form well-defined geometric defects in the gel, which act as a growth “color” for nerve fibers. The study shows that neurites (the axonal projections of neurons in the ganglion) preferentially invaded these laser-etched microchannels, aligning along them and extending in a directionally long distance [101].

In histology and immunofluorescence images, labeled neurites (β-III tubulin, a neuronal marker) appear elongated along the channels, while glial cells (S100, a Schwann cell marker) have compactly organized around these fibers, contributing to a structurally guided nerve bundle [101].

The importance of size was highlighted by the observation that the diameter of the microchannels influences cell segregation: it was initially assumed that a sub-micron caliber would selectively favor neurites vs. glia (due to the much smaller neuronal diameter), but in practice both cell types were able to penetrate the ~5 μm canaliculi, requiring further optimization of the geometry [101].

This pioneering experiment unequivocally demonstrated the role of 3D physical guidance in guiding nerve regeneration: in hydrogels with photo-inscribed microchannels, axonal growth was spatially controlled, with neurites following the trajectories imposed by the laser, unlike in uniform hydrogels where extension was chaotic [101].

Sarig-Nadir et al. point out that their laser method, which achieves cellular-scale resolution (~microns), can generate complex 3D patterns to selectively guide cells into a semi-transparent hydrogel, providing a conductive “niche” for preferential cell invasion along the created channels [101].

In addition, the material used (PEG–fibrinogen hydrogel with optical transparency) allows easy observation under a microscope of the growth process and even optical control of neural activity in the obtained networks [101].

This strategy demonstrates the potential for developing a new generation of nerve guidance tubes with internal microstructures that surpass the performance of conventional conduits [101].

In the future, photopatterned microchannels could also be integrated into constructs for the central nervous system or into 3D models of neural networks for pharmacological screening, where spatial organization is essential [101].

##### Case Study 2: Vascular Engineering—Laser Fabricated Microchannels for Graft Pre-Vascularization

Adequate vascularization is a limiting factor in the survival and integration of large tissue grafts. Cells within a 3D construct cannot survive more than ~200 μm from a functional blood vessel due to limited diffusion of oxygen and nutrients [109].

Traditional strategies use proangiogenic growth factors (e.g., VEGF) to stimulate capillary growth in situ, but their administration can cause unwanted side effects (aberrant angiogenesis, inflammation, tumorigenesis) [109].

An alternative approach, highlighted by Lee et al. (2020), consists of physically engineering microchannel networks within the implant prior to implantation, under the premise that the appropriate structure can guide the desired biological function [109].

In their model, 3D networks of microchannels were generated in a gelatin-based hydrogel, which was then implanted into animal models of ischemia, achieving rapid blood perfusion through the graft microchannels and rescuing damaged tissues [109].

This beneficial effect was associated with the polarization of macrophages towards the pro-angiogenic regenerative phenotype (M2) determined by the geometry of the channels and with the functional recovery of endothelial cells, demonstrating that the canalicular microstructure of the graft induced angiogenesis without any exogenous growth factor [109].

To create such vascular micro-networks in vitro, laser techniques offer excellent control over the geometry of the channels. Brandenberg et al. (2016) showed, for example, that 2-photon photoablation can be used to “draw” branched microchannels in biological hydrogels (type I collagen or PEG), which can then be perfused with endothelial cells to form functional blood micro-vessels [110].

In their experiment, complex channel networks (including 3D patterns such as spirals, overlapping networks, etc.) were laser-etched into a collagen hydrogel, demonstrating the feasibility of fabricating customized microfluidics directly in bulk [110].

They then infused human endothelial cells (HUVEC) through these microchannels immediately after ablation. Within ~5 days, the formation of a continuous endothelial tunnel along each channel was observed under the microscope: HUVEC adhered to the channel walls proliferated and formed a confluent layer lining the lumen, expressing markers characteristic of mature endothelium such as CD31 and VE-cadherin [110].

The importance of this result lies in obtaining a perfusable capillary network before implantation, essentially, a pre-vascularized graft. The microchannels thus populated remained open and allowed fluid perfusion, mimicking blood vessels. Song et al. (2020) emphasize in their analysis that such 3D laser techniques can produce architecturally precise hydrogel scaffolds that accommodate integrated vascular networks, essential for future clinical applications [111].

Overall, this case study demonstrates that laser patterning of hydrogels provides an efficient means to create micro-channels for endothelial cells, facilitating de novo blood vessel formation in tissue constructs. Unlike conventional vascularization methods, the laser approach allows for control of the network topology (microchannel diameter, degree of branching, interconnectivity) to meet the criteria of an ideal perfusable network (e.g., adequate diameter for flow, high density to overcome the diffusion limit, closed connections between inlet and outlet for efficient unidirectional flow) [109].

It has already been demonstrated in vivo that the implantation of gelatin hydrogels with such microchannels accelerates the reconnection of host circulation through the graft and the recovery of ischemic tissues [109].

#### 3.4.5. Advantages and Limitations

##### Advantages

A focused laser beam can create patterns with micron-level resolutions, allowing for the precise definition of microchannels, notches, or cavities in a hydrogel [101,103]. Ultrafast (femtosecond) lasers ensure energy deposition in a very small volume, resulting in uniform and well-contoured structures, without adjacent affected areas [103]. For example, femtosecond laser ablation on hydrogel contact lenses produced ~80 μm microchannels that were much sharper than those obtained with continuous CO_2_ lasers, due to the elimination of large thermal effects [103].

LDW is a direct, digital process that does not require photolithographic masks or dies. Patterns or designs can be quickly modified in software, then written onto the hydrogel in a matter of hours, significantly accelerating prototype iteration. Compared to classical fabrication (e.g., lithography + molding), laser writing offers a rapid route to creating microstructures. Nawroth et al. reported reducing the time to fabricate micropatterned substrates for a heart-on-chip by up to 60% compared to traditional lithographic approaches [105].

Laser ablation can be applied to a wide range of hydrogel materials (natural, synthetic, composite), including those that cannot be processed by photolithography (because they do not have photocrosslinkable groups) [99]. Also, the pattern geometry is not limited by the flatness of a mold, the laser can draw on both flat surfaces and curved substrates (e.g., on a contact lens) [103]. Complex microstructures have been demonstrated: spirals, 3D networks of channels, internal microcavities, all fabricated by three-dimensional laser scanning in the hydrogel volume [101,103].

In situ processing and integration with fragile systems: As a non-contact method, laser ablation can modify hydrogels already assembled into devices or even populated with cells. For example, it has been demonstrated to etch guiding channels into a hydrogel after cell seeding without significantly compromising viability, using infrared pulses that minimize cell photodamage [99]. Additionally, lasers allow the integration of hydrogels onto sensitive platforms, one study directly integrated a chemosensitive micro-hydrogel onto a fragile MEMS chip using two-photon laser writing, showing that hydrogels can be structured in situ into microsystem devices without additional complex steps [112].

##### Limitations

If the parameters are not correctly adjusted, laser energy can induce unwanted thermal effects in the hydrogel. These include burns, local charring, microcracks or gas bubbles in the material [102]. Hydrogels are often heat sensitive (they contain a lot of water); a continuous beam or long pulses can overheat the area, causing vapor bubbles to form that uncontrollably widen the channel and degrade the surrounding polymer [102]. For example, in CO_2_ laser ablation of contact lenses (continuous wave), melted edges and microbubbles were observed, due to exceeding the decomposition temperature (~360 °C) of the hydrogel during etching [102]. By switching to ultrafast pulses, many of these thermal effects can be mitigated, but caution is still needed: even femto lasers can cause deformations of the hydrogel due to the thermoresponsive properties of the polymer or the pressure of microbubbles formed during ablation [102].

Hydrogels contain water and can often be opaque or diffuse light, especially at short wavelengths. Thus, the depth to which the laser can effectively ablate is limited, typically a few tens or hundreds of microns, depending on the transparency of the material.

For large-scale production of hydrogel-based devices, LDW is not yet the ideal method. Molding remains much more industrially scalable, a review of microfluidic contact lenses notes that while laser patterning offers rapid and precise design changes, molding-based methods are better suited for mass manufacturing due to their reproducibility and shorter lead times per piece [99].

The main attributes of Laser Direct Writing, as a technique for fabricating hydrogel-based microstructures, are presented in Table 7.

## 4. Clinical Application of Hydrogel Scaffold Products

The translation of hydrogel research into practical, clinically available solutions is a primary goal for many studies in this field. While much focus remains on developing novel fabrication techniques, it is important to acknowledge that numerous hydrogel-based products are already commercially successful, demonstrating a significant and growing real-world impact in medicine. A vast number of hydrogel-based medical products, far exceeding 30, have received approval from global regulatory bodies such as the U.S. Food and Drug Administration (FDA) and the European Medicines Agency (EMA) for clinical use [116]. These products serve a wide range of applications, from wound dressings and cartilage repair to surgical sealants and drug delivery systems. Their commercial and clinical success underscores the critical need for advanced biomaterials like hydrogel scaffolds and confirms their viability as a cornerstone of modern regenerative medicine [117]. Numerous licensed hydrogel products are currently improving patient outcomes. For instance, DermiSphere hDRT is a collagen-based hydrogel scaffold designed to mimic the native extracellular matrix (ECM), in order to facilitate angiogenesis and cellular integration. It received FDA 510(k) clearance in 2018 (K183008) for the management of a variety of skin wounds, having significant potential for treating deep lesions in burn victims [118]. In South Korea, KeraHeal-Allo is an approved advanced therapy consisting of allogeneic keratinocyte cells delivered in a hydrogel sheet, specifically indicated for the treatment of second-degree burn lesions [119,120]. In orthopedics, MACI^®^ (Matrix-Induced Autologous Chondrocyte Implantation) is an FDA-approved therapy that utilizes a purified collagen hydrogel scaffold (membrane) infused with a patient’s own chondrocytes, aimed at repairing symptomatic cartilage defects in the knee [120]. While most commercial hydrogel products currently utilize conventional manufacturing methods, there is a powerful and growing trend toward integrating advanced biofabrication techniques. Companies are actively exploring the use of unconventional methods like laser-based bioprinting to create more complex and biomimetic tissues. A prominent example is the company Poietis, which is pioneering the use of laser-assisted bioprinting technology, based on the Laser-Induced Forward Transfer principle. Their research product, Poieskin^®^, is a highly sophisticated skin model comprising a synthetic dermis-like collagen hydrogel precisely infused with fibroblasts and keratinocytes by the laser printing process [121,122]. While currently used for advanced in vitro testing in the pharmaceutical and cosmetic industries, the technology showcases the clear potential for next-generation laser fabrication techniques to translate into future clinical applications for tissue repair and regeneration.

Hydrogels are widely used as debriding agents, moist dressings, and components of pastes for wound care. Numerous commercial products are available, such as Granugel^®^ (ConvaTec, London, UK), a clear, viscous hydrogel for managing partial and full-thickness wounds; Intrasite Gel^®^ (Smith & Nephew, Watford, UK), an amorphous sterile hydrogel for shallow and deep open wounds; and Purilon Gel^®^ (Coloplast, Fredensborg, Denmark), indicated for necrotic wounds and burns. These products leverage the hydrogel’s high water content to aid autolytic debridement, absorb exudate, and provide a cooling, hydrating effect, which is particularly beneficial in emergency burn treatment, as seen with the Burnshield hydrogel dressing (Levtrade International, Johannesburg, South Africa) commonly found in first aid kits [123,124,125].

One of the successful examples of hydrogels for drug delivery is the vaginal insert Cervidil^®^ for cervical ripening, which has been on the market since 1995. This controlled-release formulation contains dinoprostone in a cross-linked polyethylene oxide/urethane polymer and releases the drug over 12 h upon swelling in the moist vaginal environment [11,126]. Another licensed product is the subcutaneous reservoir system SUPPRELIN LA (Endo Pharmaceuticals Solutions Inc., Malvern, PA, USA), which delivers histrelin acetate over 12 months for the treatment of central precocious puberty. The implant is made from a non-biodegradable hydrogel of 2-hydroxyethyl methacrylate and 2-hydroxypropyl methacrylate [127]. These products demonstrate the successful translation of hydrogel technology into long-term, controlled-release therapies.

In the field of tissue engineering, patented hydrogel technologies are paving the way for commercial products. For instance, the self-assembling peptide PURAMATRIX (3-D Matrix, Inc., Tokyo, Japan) is commercialized as a nanofibrous, nanoporous hydrogel scaffold that is non-immunogenic, biodegradable, and capable of stimulating tissue ingrowth and vascularization [116]. Furthermore, European patent EP 1 664 168 B1 [116] describes a method for manufacturing porous scaffolds from a biodegradable polymer like poly(propylene fumarate) and hydrogel microparticles (e.g., cross-linked collagen or gelatin) that can contain a biologically active substance. This scaffold is designed for the treatment of skeletal defects without the need to leach out the hydrogel porogen, representing a significant step towards practical clinical application [116].

These examples demonstrate the successful translation of hydrogel technology into a wide range of clinically viable products, underscoring their importance in modern medicine and highlighting the potential for advanced fabrication techniques to contribute to future innovations [116].

## 5. Challenges

The use of advanced laser technologies to fabricate hydrogel scaffolds promises unprecedented resolutions and complex geometries that are impossible to achieve with conventional methods. However, these benefits come with numerous material and technical challenges. High precision typically involves slow manufacturing speeds, limited scalability, and expensive equipment [128]. Furthermore, the compatibility of hydrogels with laser processes is not always guaranteed as many biomaterials require chemical modifications to become photosensitized or withstand process conditions [45]. The following critical analysis discusses the major difficulties associated with each laser method (2PP, LIFT, SLM/SLS, LDW) in the context of hydrogels, highlighting impediments of a material (photochemistry, thermal stability), technological (yield, resolution, equipment complexity) and functional (structural integrity and cellular viability of the obtained scaffolds) nature.

Two-Photon Polymerization. This 2PP technology offers unique sub-micron resolution, but suffers from significant limitations related to speed, size of achievable structures, and usable materials [45]. Being a serial process (voxel by voxel), 2PP has a very low throughput; writing a structure of just a few millimeters can take hours to days [45]. This limited speed makes it difficult to obtain large volumetric scaffolds and raises industrial scalability issues. At the same time, the necessary equipment (high-power femtosecond lasers, optical systems, and nano-metric positioning platforms) impose extremely high acquisition and operating costs [128]. Another difficulty is the limited range of biocompatible photopolymerizable materials: most natural hydrogels must be functionalized (e.g., with methacrylate groups) in order to be crosslinked by 2PP [1,45]. Even then, unreacted monomers or residual photoinitiators with cytotoxic potential may persist in the fabricated structures, affecting cell viability [45].

Laser-Induced Forward Transfer. LIFT allows for contactless deposition of cell-based bio-inks but has difficulties in achieving large volumetric constructs. The transfer of hydrogel droplets is performed point by point, which leads to a relatively slow process and is difficult to scale up to centimeter-sized structures [79]. In practice, LIFT is more suitable for small constructs or fine 2D/3D patterns, printing a complete organ being inefficient due to the very long time required. This throughput limitation requires maintaining sterile conditions for long periods and increases operational complexity. On the other hand, LIFT systems are complex and expensive, involving high-energy pulsed lasers, precise focusing optics, fine alignment between substrates and environmental control (to prevent the bio-ink layer from drying out). Interventionary studies involving animals or humans, and other studies that require ethical approval must list the authority that provided approval and the corresponding ethical approval code. This restricts the use of LIFT to specialized laboratories. Each new bio-ink formulation requires careful calibration of parameters (laser energy, viscosity, donor layer thickness, etc.), and there are still no generally valid standards—the trial-and-error effort being substantial for each application. From a functional point of view, the creation of thick and viable tissues by LIFT faces obstacles: layer-by-layer printed structures can suffer from the lack of complete fusion between droplets and insufficient diffusion of nutrients inward, leading to cell death in the center of the construct. Also, the integration of vascular networks remains problematic—attempts to print micro-capillaries (<100 μm) have shown instability, with channels collapsing in the absence of a supporting matrix or immediate perfusion. Another inherent disadvantage of LIFT is the generation of shock waves upon droplet ejection: the sudden increase in temperature and pressure in the small volume of the donor layer produces shock waves that can destabilize the jet and even damage the transferred cells. Solutions such as printing in a partial vacuum environment or using ultra-short pulses to attenuate these shock waves have been proposed [129].

The main limitation of SLS for bioactive applications is the high process temperature (typically ~100–200+ °C for polymers and much higher for metals). Under these conditions, living cells or sensitive growth factors cannot be directly printed: any biological component introduced into the powder would be locally exposed to temperatures that destroy cells or denature proteins [84,90].

Although SLS is a promising technique, its compatibility is not universal, especially for materials intended for hydrogels. The process requires fine powders with excellent flow properties and thermal stability. For polymers forming hydrogels, meeting these criteria simultaneously is difficult, and common routes to obtain fine powders frequently introduce complications.

The most common method, milling, generates particles with irregular shapes and broad particle size distributions. These characteristics favor agglomeration and uneven layering, which results in defects in the final part. Ideally, spherical powders, obtained by atomization or controlled precipitation, would be used; however, these processes are expensive and may involve toxic solvents, limiting their applicability in some contexts. To compensate for the poor flow of irregular powders, additives are sometimes used (e.g., fumed silica as a “flow agent”), which help form a thin and uniform layer—an essential condition for the success of SLS [90].

Compared to stereolithography or electrospinning, SLS does not excel at microdetailing below ~100 μm. The minimum feature size is constrained by the laser spot diameter (typically 200–500 μm for CO_2_ lasers) and the powder particle size (≈50–100 μm). As a result, very sharp edges, corners, and features <~0.5 mm tend to be blurred or impossible to reproduce faithfully [85].

Narrow thermal window and collateral damage. Laser direct writing/ablation in hydrogels is sensitive to energy dosage and pulse durations; exceeding the local threshold produces heat-affected zones, charring, microcracks and cavitation bubbles that can distort fine geometries or damage nearby cells/biomolecules [43,101]. Ultrafast pulses (fs/ps) significantly reduce thermal diffusion, but do not eliminate it photoacoustic shock and bubble dynamics remain critical; in continuous or long-IR regimes (e.g., CO_2_), photothermal coupling is pronounced and widens channels by melting/evaporation, with the risk of polymer network degradation [43,101,130].

While each laser-based bioprinting technique offers unique advantages, they are also accompanied by distinct limitations that influence their suitability for specific applications. A summary of the primary challenges associated with each method is presented in Table 8.

## 6. Conclusions and Future Directions

The advancement of laser-based fabrication techniques has significantly expanded the possibilities for engineering hydrogel scaffolds, with each method offering unique advantages tailored to specific biomedical applications.

Two-photon polymerization stands out due to its unparalleled sub-micron resolution, enabling the fabrication of highly intricate 3D microstructures ideal for mimicking cellular microenvironments. However, its slow processing speed and high costs limit its use to small-scale, high-precision applications. In contrast, laser-induced forward transfer excels in nozzle-free, high-resolution bioprinting of living cells and bioinks with minimal damage, making it particularly valuable for creating cell-laden constructs. Yet, its throughput remains limited for large tissue fabrication.

Recent pioneering work, such as that by Gehre et al. (2024), has demonstrated the remarkable potential of laser-based techniques not only for fabrication but also for post-printing modification to guide cell behavior [132]. In their study, they employed photosensitized two-photon ablation to precisely sculpt channels within a 3D hydrogel, effectively guiding the formation of bone cell networks. This exemplifies a powerful trend moving beyond mere printing towards the active manipulation of the biofabricated microenvironment [132].

Building on this, the future of laser-based bioprinting, particularly 2PP, lies in its unique ability to create complex, mechanically nuanced, and biomimetic microstructures with sub-micron resolution, otherwise unattainable with other biofabrication technologies. This is crucial for replicating the hierarchical architecture of native tissues, such as the osteons in bone, or the intricate vascular networks essential for nutrient perfusion in large constructs. Future perspectives should explore the integration of 2PP with other bioprinting modalities (e.g., extrusion-based printing of bulk matrices) in a multi-modal approach. Furthermore, the development of novel bioresins that are highly biocompatible for cell encapsulation during the 2PP process itself, while also offering tailored mechanical and biochemical properties, remains a key research direction. The work discussed by the reviewer provides a compelling roadmap for how laser systems can evolve from passive fabrication tools to active, dynamic platforms for guiding tissue morphogenesis in situ [132].

SLM/SLS laser is advantageous for producing mechanically robust, porous scaffolds from thermoplastic powders, but its high-temperature processing restricts direct use with sensitive hydrogels and biomolecules. Meanwhile, laser direct writing offers precise subtractive patterning of pre-formed hydrogels, allowing for microchannel creation and surface modifications that guide cell behavior, though its depth penetration and scalability are constrained.

While 2PP and LIFT are better suited for soft, cell-compatible hydrogels requiring fine detail, SLM/SLS is optimal for sturdier, load-bearing scaffolds. LDW complements these methods by enabling post-fabrication modifications. Future progress lies in hybrid approaches that combine these techniques for instance, using SLS for structural support and LIFT for cell integration to overcome individual limitations. Advancements in laser technology, biocompatible materials, and automation will be crucial for translating these methods into scalable, clinically viable solutions. By leveraging their complementary strengths, laser-based fabrication can drive the next generation of personalized regenerative therapies.

**Figure 6:**
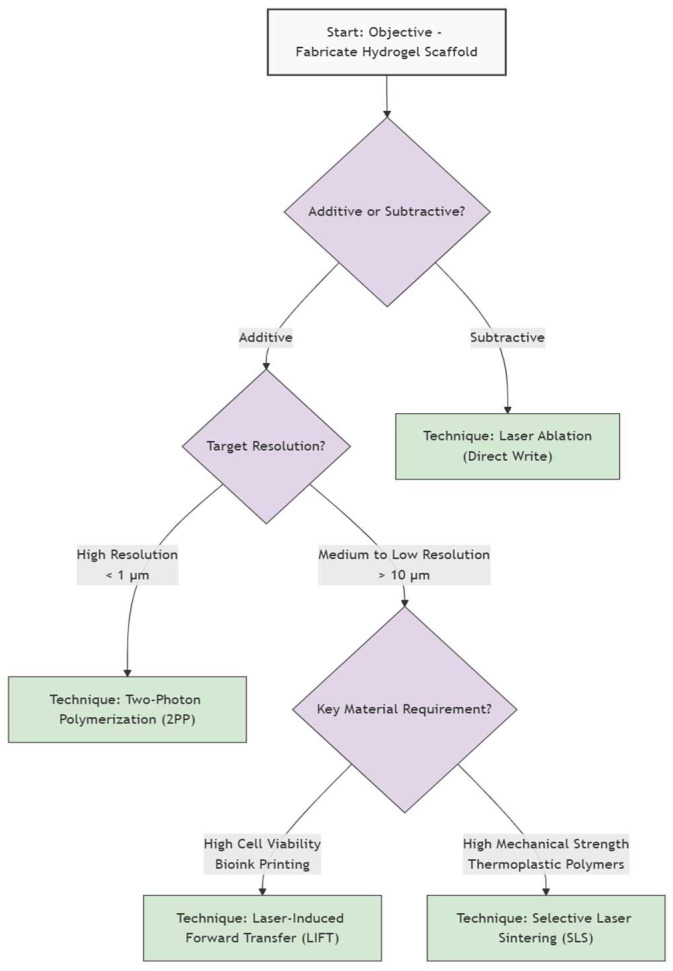
Technology roadmap for laser-based hydrogel fabrication. This flowchart visualizes the decision-making process for selecting a laser fabrication technique for hydrogel scaffolds, beginning with the fundamental choice between additive and subtractive manufacturing. The subsequent steps guide users to the optimal technique (e.g., Two-Photon Polymerization, Laser-Induced Forward Transfer, Selective Laser Sintering/Melting, or Laser Direct Writing) by considering specific application requirements, including desired resolution and material type.

A technology roadmap in the form of a decision tree (Figure 6) can guide researchers in selecting the most suitable laser-based fabrication technique for hydrogel scaffolds based on key application requirements such as resolution, material type, and cell viability.

A future area of interest might be volumetric 3D printing. Volumetric 3D printing (VBP) is widely considered by researchers and industry experts to be a genuine paradigm shift in additive manufacturing, not just an incremental improvement. The enthusiasm for this technology is rooted in its revolutionary advantages over traditional layer-by-layer printing. VBP offers unprecedented speed, by solidifying an entire volume simultaneously, eliminating the fundamental speed constraints of conventional scanning methods [133]. This makes it ideal for rapid prototyping and printing large objects. Additionally, the technology produces objects with superior surface quality because it is free from the layer lines and stair-stepping artifacts inherent in other systems [134]. Finally, VBP’s ability to print without physically touching the material makes it particularly exciting for bioprinting and soft matter engineering, as it allows for the encapsulation of delicate living cells and sensitive proteins without mechanical damage [135].

Despite its potential, the scientific community maintains a realistic perspective on the current challenges and limitations of VBP. A significant hurdle is caused by material constraints, as the available resins must be optically transparent and possess specific photo-reactivity properties [135]. Furthermore, researchers have noted geometric and mechanical limitations, such as the difficulty in creating large overhangs or complex internal voids without support structures [135]. Another challenge is the complexity of the hardware and software required to calculate the exact light patterns from multiple angles, which makes the technology less accessible than other, better established methods [136].

Rather than being a panacea for all 3D printing, VBP’s immediate future lies in high-impact, niche applications, where its unique advantages are indispensable. These include biomedical engineering, such as printing patient-specific anatomical models for surgical planning and creating cell-laden constructs for tissue engineering [136]. VBP is also a powerful tool for advanced optics, where it can fabricate lenses and light guides with flawless clarity and smooth surfaces, as well as in customized consumer products for rapid manufacturing of unique, high-quality designs [135]. Overall, the consensus is that VBP is a transformative technology with the potential to redefine speed and quality standards in additive manufacturing, but its widespread adoption depends on future breakthroughs in material science and simplification of the printing process [135,136].

## Figures and Tables

**Table 1:** Hydrogel classifications and properties.

Hydrogel Type	Advantages	Disadvantages	References
Natural hydrogels	Inherent biocompatibility and bioresorbability; Biochemically similar to the extracellular matrix (ECM); Biodegradable, with non-toxic byproducts (e.g., Collagen, hyaluronic acid (HA), alginate).	Lack of intrinsic cell-adhesive ligands, which may require modification; Enzymatic degradation rate can be unpredictable.	[6,7,8,9,10,11,12]
Synthetic hydrogels	Offer greater control over chemical composition, mechanical properties, and durability; Degradation rate is more predictable and tunable; (e.g., Polyethylene glycol (PEG), polyvinyl alcohol (PVA), polyacrylamide (PAAm)).	Generally bioinert and lack cell-recognition signaling, requiring functionalization for cell adhesion.	[6,7,8,9,10,11,12]
Hybrid hydrogels	They combine the advantages of both natural and synthetic types: Biocompatibility and cell binding sites from natural polymers. Mechanical strength and tunable structure from synthetic polymers. Examples: Chitosan/PVA blends, alginate modified with PEG.	Many times it can be hard to tune, based on applications.	[6,7,8,9,10,11,12]

**Table 2:** Mechanical properties of hydrogels for medical applications.

Hydrogel Type	Relevant Properties and Characteristics	Young’s Modulus (Elasticity Modulus) Range	Associated Tissue Mimicry	Key References
Chemically Crosslinked Hydrogels (e.g., PEGDA, Alginate)	Tunable stiffness and mechanical resistance via crosslink density, polymer concentration, and molecular weight. Softer hydrogels are suitable for soft tissue, while more rigid ones can support hard tissue.	~0.1 kPa to >100 kPa (for soft tissues); can reach into the low MPa range for hard tissue mimics.	General soft (e.g., brain, fat, muscle) and hard tissues (e.g., bone).	[25,26]
Photo-crosslinked Gelatin Methacrylate (GelMA)	Excellent bioactivity (contains cell-adhesive RGD motifs). Supports chondrocytes and new ECM production. Stiffness is tuned by UV exposure time, photoinitiator concentration, and methacrylation degree.	~1 kPa to 100 kPa is most common for cell culture. The statement “a few MPa” is high but possible with very high polymer concentration and crosslinking, though it exceeds typical values for cartilage.	Cartilage (modulus ~0.1–1 MPa), but also widely used for various soft tissues like blood vessels and muscle.	[27,28]
Double Network (DN) Hydrogels	Comprise two interpenetrating polymer networks: a rigid, brittle first network and a soft, ductile second network. This structure dissipates energy under load, leading to high toughness and fracture resistance.	Compressive strength can indeed reach tens of MPa (e.g., 10–40 MPa). Elastic modulus typically ranges from ~0.1 MPa to 1.0 MPa, which is a direct match for native cartilage.	Cartilage tissue (articular cartilage modulus: ~0.2–1.0 MPa in compression). Excellent mimic due to high water content and toughness.	[29]
Polymer–Ceramic Composites (e.g., GelMA-HAp, PEG-HAp)	Created by mixing polymers with inorganic materials like hydroxyapatite (HAp) or calcium carbonate to increase rigidity and provide osteoinductive signals for bone tissue engineering.	Wide range: 10 MPa to 2 GPa+. The stiffness depends heavily on the ceramic content (e.g., from 10% to 70% by weight). The trade-off with water absorption is important, as ceramics are hydrophilic but not swellable.	Hard tissues, such as bone (cortical bone modulus: ~15–20 GPa; trabecular bone modulus: ~0.1–2 GPa). Composites aim to approach the lower end of this range.	[30]
Porous PVA Hydrogels (via SLS)	Created via selective laser sintering (SLS), a 3D printing technique that creates a periodic-porous structure. This porosity is critical for nutrient waste exchange and cell migration. PVA is biocompatible and supports osteoblastic cell adhesion.	The modulus is not specified in your text, which is common, as it varies greatly with porosity and structure. However, for bone applications, targets are typically in the MPa to low GPa range. The key property is often the compressive strength and the scaffold’s architecture.	Bone tissue, specifically for creating scaffolds that mimic the microporosity and mechanical function of trabecular bone.	[31,32]

**Table 3:** Principles of the laser-based methods.

Method	Principles
Two-photon polymerization	It uses two-photon absorption of near-infrared radiation to induce polymerization in a photosensitive material only at the laser’s focal point. This allows for direct 3D writing without a mask and at a sub-diffraction limit resolution.
Laser-induced forward transfer	A pulsed laser is focused on an absorbing layer, creating a pressure wave that propels a droplet of a donor material (bio-ink) toward a collector substrate. It is a nozzle-free, direct deposition technique.
Selective laser sintering/melting	A high-power laser selectively fuses fine powder particles, layer by layer, to create a 3D structure. The heat consolidates the powder physically without chemical additives.
Laser ablation	A focused laser is used to precisely remove material from a hydrogel with high precision, creating patterns, grooves, or internal channels.

**Table 4:** Key parameters and characteristics of Two-Photon Polymerization for high-resolution additive manufacturing.

Parameter	Summary	References
Resolution	Extremely high resolution (<100 nm to <1 micron); Surpasses the optical diffraction limit via nonlinear absorption; Ideal precision for mimicking cellular and subcellular environments.	[56,57]
Speed	Slow, voxel by voxel processing speed, due to the serial writing process; Limited to small scale, high precision applications.	[58,59]
Suitable Materials	Compatible with photosensitive materials either natural (GelMA, hyaluronic acid methacrylate) or synthetic (PEGDA, PEGDMA); biocompatibility and cell binding sites from natural polymers; Materials must be functionalized with photopolymerizable groups (e.g., acrylates, methacrylates.	[28,60]
Cell Compatibility	Can create cell-laden scaffolds; Conventional, cytotoxic UV initiators (e.g., Irgacure 2959) are commonly replaced by biocompatible water-soluble initiators (e.g., LAP, Eosin Y); High cell viability (>85–90%).	[61,62]
Key Advantages/Limitations	Unparalleled resolution; Real 3D free form writing; Slow writing speed; High setup cost; Material selection limited to photopolymers.	[56]

**Table 5:** Key parameters and characteristics of Laser-Induced Forward Transfer for nozzle-free bioprinting.

Parameter	Summary	References
Resolution	High spatial resolution (10–100 μm); Nozzle-free, picolitre precision bioink deposition; Influenced by spot size, laser energy and ribbon properties.	[68]
Speed	High droplet ejection rate (order of kHz)—experimentally about 5 kHz; Volumetric throughput limited by the droplet placement accuracy requirements.	[81]
Suitable Materials	Wide variety of either low or high viscosity hydrogels (e.g., alginate, collagen, fibrin, Matrigel, cell spheroids); Material must form a uniform layer with the ribbon (donor substrate).	[68,82]
Cell Compatibility	Excellent for living cells; Gentle process, cells are shielded by a laser-absorbing interlayer (e.g., gold, titanium); Cell viability > 90–95%.	[82,83]
Key Advantages/Limitations	Nozzle free (avoids clogging); High cell viability; Allows for use of high viscosity materials and cell aggregates; Limited by the volume in the ribbon, possibility of satellite droplets and ribbon manufacturing complexity.	[68,82]

**Table 6:** Key parameters and characteristics of Selective Laser Sintering for fabricating porous, acellular scaffolds.

Parameter	Summary	References
Resolution	Limited resolution (>100 μm); Limited by spot diameter, powder particle size (typically 50–100 μm) and thermal diffusion, potentially leading to blurring of features.	[96,97]
Speed	Fast, layer by layer laser scanning; Significantly slowed by additional steps, i.e., pre-heating, powder recoating, cooling.	[97,98]
Suitable Materials	Thermoplastic polymers (e.g., PCL, PVA, PLA); Metals; Ceramics in powder form (need thermal and flow properties compatible with sintering).	[97,99]
Cell Compatibility	Incompatible with direct cell printing; High temperature, often exceeding the polymers’ melting points (~60 °C for PCL).	[96,100]
Key Advantages/Limitations	Makes complex, mechanically robust scaffolds without supports (the powder acts as support); High temperatures do not allow for direct cell use; Rough surface finish; Powder handling can pose additional challenges.	[96,97]

**Table 7:** Key parameters and characteristics of Laser Direct Writing for direct crosslinking of hydrogel-based constructs.

Parameter	Summary	References
Resolution	Patterns with micron-level resolution (10–100 μm); Precise crosslinking enabled by ultrafast lasers (heat diffusion is reduced).	[113]
Speed	Fast for prototyping; Serial process, but faster than 2PP; Highly dependent on laser power, scanning speed and material photosensitivity.	[114]
Suitable Materials	Thermoplastic polymers (e.g., PCL, PVA) Primarily photopolymerizable hydrogels (e.g., GelMA, PEGDA); Materials must have strong crosslinking capabilities.	[113,115]
Cell Compatibility	High cell viability is possible using near-IR femtosecond lasers (heat damage is minimized); Microchannel growth is enabled, guiding cell growth.	[113,115]
Key Advantages/Limitations	Direct, digital, mask-free process for creating 3D microstructures within hydrogels; Gentle on cells; Limited depth penetration (caused by light scattering); Scalability issues due to serial writing.	[113,114]

**Table 8:** Key limitations and challenges of laser-based bioprinting techniques.

Technique	Primary Limitations	Key References
Two-Photon Polymerization (2PP)	Very slow printing speed; High cost; Limited to photopolymerizable materials; Potential cytotoxicity of photoinitiators, unless properly optimized.	[56,58,131]
Laser-Induced Forward Transfer (LIFT)	Limited volumetric throughput for large constructs; Potential for satellite droplet formation; Cost and complexity of laser systems.	[96,97]
Selective Laser Sintering (SLS)	High processing temperatures prevent direct cell printing; Limited resolution (>100 μm); Rough surface finish; Limited to thermoplastic powders with specific flow and sintering properties.	[31,96,97]
Laser Direct Writing (LDW)	Limited resolution (>100 μm); Limited depth penetration due to light scattering; Serial process limits scalability; Requires photopolymerizable hydrogels.	[113,114]

## Data Availability

No new data were created or analyzed in this study.
